# Developing Climate-Resilient, Direct-Seeded, Adapted Multiple-Stress-Tolerant Rice Applying Genomics-Assisted Breeding

**DOI:** 10.3389/fpls.2021.637488

**Published:** 2021-04-15

**Authors:** Nitika Sandhu, Shailesh Yadav, Margaret Catolos, Ma Teresa Sta Cruz, Arvind Kumar

**Affiliations:** ^1^Rice Breeding Platform, International Rice Research Institute, Metro Manila, Philippines; ^2^School of Agricultural Biotechnology, Punjab Agricultural University, Ludhiana, India; ^3^International Rice Research Institute South Asia Regional Centre, Varanasi, India

**Keywords:** direct seeded, genomics, multiparent, QTL/genes, pyramiding, *Oryza sativa*

## Abstract

There is an urgent need to breed dry direct-seeded adapted rice varieties in order to address the emerging scenario of water–labor shortage. The aim of this study was to develop high-yielding, direct-seeded adapted varieties utilizing biparental to multiparental crosses involving as many as six different parents in conventional breeding programs and 12 parents in genomics-assisted breeding programs. The rigorous single plant selections were followed from the F_2_ generation onwards utilizing phenotypic selection and quantitative trait locus (QTL)/gene-based/linked markers for tracking the presence of desirable alleles of targeted QTL/genes. In conventional breeding, multiparent lines had significantly higher yields (2,072–6,569 kg ha^−1^) than the biparental lines (1,493–6,326 kg ha^−1^). GAB lines derived from multiparent crosses had significantly higher (3,293–6,719 kg ha^−1^) yields than the multiparent lines from conventional breeding (2,072–6,569 kg ha^−1^). Eleven promising lines from genomics-assisted breeding carrying 7–11 QTL/genes and eight lines from conventional breeding with grain-yield improvement from 727 to 1,705 kg ha^−1^ and 68 to 902 kg ha^−1^, respectively, over the best check were selected. The developed lines may be released as varieties/parental lines to develop better rice varieties for direct-seeded situations or as novel breeding material to study genetic interactions.

## Introduction

Rice is mainly cultivated under anaerobic conditions and primarily adapted and evolved under these conditions (Kumar and Ladha, 2011). A shortage of water and labor input makes rice production through transplanted rice (TPR) more expensive, less profitable, and unsustainable (Farooq et al., 2011). The estimates specify that the water requirement for irrigation by 2025 could be approximately 561 km^3^ for a low-demand scenario and 611 km^3^ for a high-demand scenario (Kumar et al., 2005). The demand for water in various non-agricultural sectors in the country reduces its availability for agriculture; as a result, the agricultural sector is likely to face another 10–15% reduction in irrigation water by 2025 (Boretti and Rosa, 2019). With 90% of the world's rice grown and consumed in Asia and the large water requirements of conventional transplanted rice cultivation systems, it is evident that this water scarcity will severely affect rice production in this continent. The water scarcity situation will lead to rice cultivation with less water (Wang et al., 2002).

Cultivation of dry direct-seeded rice (DDSR) helps in avoiding three basic operations: puddling, transplanting, and maintaining standing water. DDSR cultivation in saturated soil has been widely adopted in southern Brazil, Chile, Cuba, Venezuela, some of the Caribbean countries, and in certain areas of Colombia (Fischer and Antigua, 1996). Currently, DDSR is becoming more popular in South Asia, South East Asia, and, to some extent, in West Africa. The cultivation of DDSR is being practiced with several modifications of tillage or land preparation and crop establishment (CE) with a site-specific package, but has not gained the required popularity because of one or two unsolved deficiencies. In addition to higher economic returns, DDSR crops vis-à-vis the transplanted (TPR) system are faster and easier to plant, have a shorter duration, are less labor intensive, and consume less water (Bhushan et al., 2007). The mechanized DDSR cultivation system has been estimated to provide potential irrigation water savings of 40 cm ha^−1^, labor savings of 25 person-days ha^−1^, energy savings of 1,500 MJ ha^−1^, a reduction of GHG (green house gas) emissions of 1,500 kg CO_2_ equivalent ha^−1^, and a yield increase of 0.5 t ha^−1^ with an increased net economic return of USD 50 ha^−1^ in most rice-growing countries (Kumar and Ladha, 2011).

The development of DDSR varieties depends on several factors, such as selection of traits, identification, and introgression of genomic regions associated with those particular traits of interest. The traits that are expected to play an important role in providing yield stability and adaptability under direct-seeded conditions include anaerobic germination (ability to germinate under water, Ghosal et al., 2019), early uniform seedling emergence (Dixit et al., 2015; Sandhu et al., 2015), vegetative vigor, root phenotypic plasticity, proper nutrient uptake (Sandhu et al., 2015), and lodging resistance (Yadav et al., 2017; Sandhu et al., 2019b; Subedi et al., 2019). The biotic stress (disease and insect) resistance includes blast (Qu et al., 2006), brown spot (Sato et al., 2008), bacterial blight (Ullah et al., 2012), sheath blight, brown planthopper (Jairin et al., 2007), gall midges (Nair et al., 1996), and nematode resistance (Das et al., 2011).

Existing rice varieties are not specifically developed for direct-seeded ecosystems. However, some earlier developed rice varieties for transplanted conditions have been adapted to direct-seeded conditions exhibiting a yield decline under direct-seeded conditions. The newly developed drought-submergence-tolerant, rainfed-adapted rice varieties have shown good performance in India and Nepal, providing a yield advantage of 1.0 t ha^−1^ over the currently grown drought-submergence susceptible varieties (Sandhu et al., 2019a). Varieties such as Sahbhagi dhan, DRR 42, DRR 44, CR dhan 201, CR dhan 203, and Swarna Shreya (Sandhu and Kumar, 2017) developed by the International Rice Research Institute (IRRI) and genomics-assisted derived CR dhan 801 varieties have become highly popular in India and are reported to be cultivated on at least an area of 0.8 m ha. Similarly, in Nepal, the rice varieties Sukha dhan 3 and Sukha dhan 5 (Sandhu and Kumar, 2017), and the genomics-assisted derived lines Bahuguni dhan-1 and Bahuguni dhan-2 (Sandhu et al., 2019a) have become highly popular and contributed to the increased rice production in Nepal. Based on these achievements, the development of direct-seeded adapted rice varieties could provide opportunities for a significant increase in rice productivity in rice-growing countries.

Breeding methods such as QTL/gene pyramiding and multiparent application have been reported to be effective in developing resistant/tolerant varieties against biotic and abiotic stresses (Koide et al., 2009; Bandillo et al., 2013; Sandhu et al., 2019b). Conventional breeding is an efficient approach for the development of novel genetic variants (Breseghello and Coelho, 2013). The conventional breeding approach suffers from the problem of linkage drag, leading to the transfer of undesired traits closely linked with traits of interest. The use of molecular markers in breeding programs improves the efficiency of traditional breeding by enabling breeders to select trait-linked molecular markers (Collard and Mackill, 2008). The existence of abundant genetic diversity, genomic variation in the rice gene pool, and availability of modern genomic tools/techniques provides opportunities to choose suitable donors that are free from undesirable linkage and to dissect the quantitative nature of associated traits of interest. Most of the rice landraces perform better under various nutrient-deficient conditions (Wissuwa and Ae, 2001). Screening of diverse genotypes under direct-seeded conditions by assessing various biometrical traits, such as root architecture, plant height, and yield-related traits, will help to identify genotypes with improved yield, better root traits, higher nutrient uptake, higher nutrient utilization efficiency, and better plant type traits.

However, identification of donors, traits, and QTL for traits increasing rice yield under direct-seeded conditions and their introgression in elite genetic backgrounds using advances in genomics-assisted breeding (GAB) present a unique scenario to achieve significant yield advantages under direct-seeded conditions. The present study was undertaken to develop new direct-seeded adapted rice varieties utilizing the identified donors, genes/QTL, and their introgression in elite genetic backgrounds, compare the performance of bi-parental and multi-parental developed lines under dry direct-seeded conditions, and compare the performance of multiparent developed lines through conventional breeding and GAB approaches.

## Materials and Methods

The study on the development of direct-seeded adapted rice lines through conventional and GAB approaches began at the International Rice Research Institute (IRRI), Los Baños, Laguna (Philippines) in 2009, with the identification of suitable traits and genomic regions associated with the traits improving grain yield and adaptability of rice under dry direct-seeded cultivation conditions.

### Selection of Parents for the Conventional Breeding Program

The parents used in the conventional breeding program included widely grown high-yielding rice varieties under the transplanted system, drought-tolerant lines adapted to upland and shallow lowland conditions, and direct-seeded adapted breeding lines possessing traits required to increase rice adaptability to direct-seeded conditions. The conventional breeding program involved two, three, four, five, and six parent crosses. Detailed information on breeding lines/varieties used and the traits they possessed is presented in Table 1.

**Table 1:** Detailed information on trait characteristics and parents used to develop breeding lines in conventional breeding program.

**Cross type**		**Parent used in the crossing program**	**Traits/characteristics**
Biparental	Popular released rice varieties	Abhaya, Agami M 1, Apo, Arang, B 1050 D-KN-1-1-1-1-3, Banda, Bunga Mehu, Beaq Pendjalin, Boder, Benong 130, BR28, BR29, Bea Balok Loas, Benong 130, Cicih Gedu, Glenteng, Gendjah Ratji, Gropak gede 106, IR64, IRRI 148, IRRI 163, IRRI 176, IRBB60, Intok, Kali Aus, Kam, Ketan Gadjih Sungut, Ketan merah, Khilak, KHO487-4, Lakhon, PSBRc 10, PSBRc 28, PSBRc 82, MTU1010, MR 219, NSICRc 222, Nogo Bele 2, Mahsuri, Swarna, Samba Mahsuri, Saro 5, Tjempo Krembung, TME 80518, Vandana, Vasistha, Sabitri, UPLRi 5, UPLRi7	**Duration:** Early maturing, medium duration, late duration **Plant height:** semi-dwarf, Adaptation: rainfed, upland direct seeded **Biotic resistance:** bacterial late blight, blast, brown plant hopper, gall midge **Root traits improving nutrient uptake under direct-seeded cultivation and responsiveness to fertilizer application:** Phenotypic root plasticity, nodal root number, root hair length, root hair density **Seedling establishment traits:** early vigor, early and uniform germination **Grain yield and grain quality:** high-yielding irrigated rice varieties, grain yield under drought, grain yield under direct-seeded cultivation conditions, possessing good grain type and grain quality characteristics, popular rice varieties in different countries. **Nematode tolerance**, **Lodging resistance**
	Improved breeding lines	HHZ 5-DT20-DT2-DT1, HHZ 8-SAL 6-SAL 3-Y2, IR04N114, IR04A212, IR05N412, IR06N119, IR06A144, IR06N155, IR07N112, IR07L270, IR08L119, IR08L126, IR08L181, IR08L183, IR08L216, IR08L217, IR09N538, IR09L120, IR09L179, IR09L204, IR09L224, IR09A228, IR09L272, IR09L303, IR09L337, IR09L342, IR09L336, IR09L337, IR09L342, IR10A134, IR10L146, IR10F188, IR10L411, IR09N534, IR11L152, IR11L186, IR11L261, IR 106542, IR 12979-24-1, IR 55419-04, IR 47761-27-1-3-6, IR 67966-44-2-3, IR 67962-84-2-2-2, IR 72022-46-2-3-3-2, IR 73012-137-2-2-2, IR 74371-70-1-1, IR 81039-B-173-U 3-3, IR 81063-B-94-U 3-1, IR 81896-B-B-182, IR 87707-445-B-B-B, IR 90265-B-551-1, IR 90266-B-228-1, IR 91648-B-114-B-1-B, PR 30245-10-414, PR 35805-B-9-2-3-2-3, PR 37866-1B-1-4, PR 37942-3B-5-3-2, PR 37951-3B-37-1-2, PR 37139-3-1-3-1-2-1,WAB 878-6-37-4-4-P2-HB, WAB 880-1-27-9-2-P1-HB	
Triparental	Popular released rice varieties	Abhaya, Benong 130, BRRI DHAN 52, Bulu Gendjah, IRRI 154, IRRI 176, IRBB 23, Ma Zhan (Red), PR 30138-35-2, Q 74, Rathu Heenati, Tadukan, WS 91	
	Improved breeding lines	B 1050 D-KN-1-1-1-1-3, HHZ 8-SAL 6-SAL 3-Y2, IR04N114, IR08L152, IR08L181, IR08N194, IR09L179, IR09L204, IR09L303, IR09L336, IR09L337, IR11L269, IR11L101, IR11L184, IR 111249, IR 111250, IR 103569, IR 43070-UBN 511-2-1-1-1, IR 55423-01(NSICRc 9), IR 71700-247-1-1-2, IR 77298-14-1-2-10, IR 84984-83-15-18-B-B, IR 91648-B-1-B-3-1, IR 96322-34-223-B, IR09N538, IR BB 60, IR 93312-30-101-20-3-66-6, IRRI 154, IR 94225-B-82-B, IR 91648-B-32-B, IR 97153-B-123-B, IR 97152-B-280-B, IR09L342	
Quadraparental	Popular released rice varieties	Bea Balok Loas, Benong 130, Beaq Pendjalin, Bunga Mehu, IR 87707-446-B-B-B, MR 219, MTU 1010	
	Improved breeding lines	B 1050 D-KN-1-1-1-1-3, IR05N412, IR06N119, IR06N155, IR08N121, IR08L216, IR09L120, IR09L179, IR09L204, IR09A228, IR09L303, IR09L336, IR09L337, IR10N108, IR10N237, IR 67966-44-2-3, IR 87707-446-B-B-B, IR 86929-B-377-49-42, IR 88288-10-4-1-4, IR 90266-B-228-1, IR 90265-B-551-1, IR 90265-B-551-1, IR 90266-B-228-1, HHZ 8-SAL 6-SAL 3-Y2, PR 30245-10-414, PR 35805-B-9-2-3-2-3, PR 37139-3-1-3-1-2-1, PR 37866-1B-1-4, PR 37951-3B-37-1-2	
Pentaparental	Popular released rice varieties	COL 1, IRRI 148, IRRI 154, IRAT 120, Kali Aus, PSBRc 82, Suakoko, Swarna, Vandana, IR 87707-446-B-B-B	
	Improved breeding lines	M 312 A-74-2-8-8, P 2057-F4-88-3-1	
Hexaparental	Popular released rice varieties	Dular (ACC 32561), IR 74371-70-1-1, IRRI 148, IRRI 154, Kali Aus, Kalinga III, IR 87707-446-B-B-B, PSBRc 82, Sambha Mahsuri, Swarna, UPL Ri 7, Vandana	
	Improved breeding lines	WAB 880-1-27-9-2-P1-HB	

### Conventional Breeding Program

**Figure 1:**
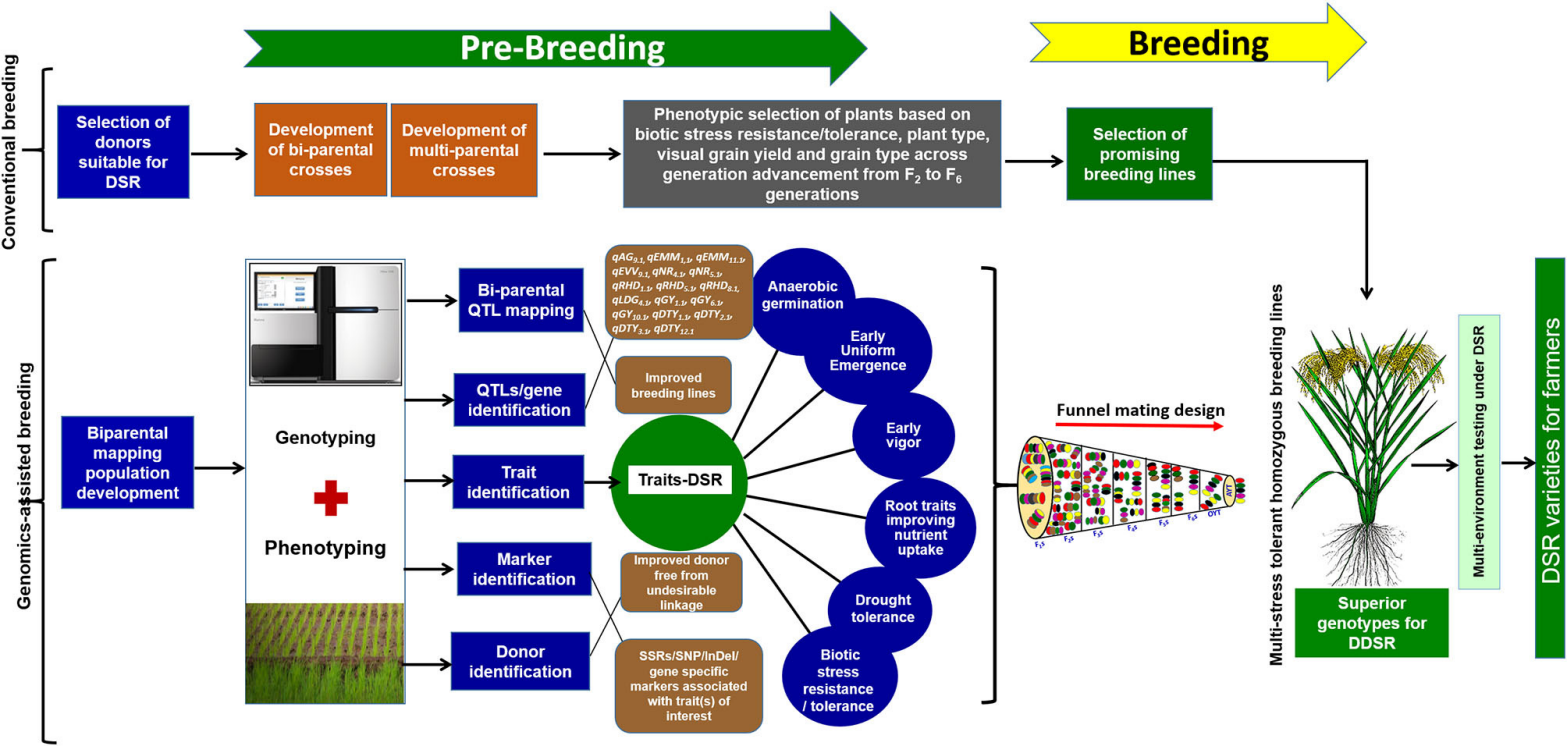
Detailed schematic representation of the pre-breeding steps, use of genomics-assisted breeding following funnel mating design to assemble the targated QTL/genes to develop multi-stress-tolerant homozygous breeding lines suitable for direct-seeded cultivation conditions.

The conventional breeding hybridization program to develop suitable adapted breeding lines involving the parents presented in Table 1 was initiated in the dry season (DS) of 2009. New crosses were included in each successive season. The F_1_s were self-pollinated and phenotypic selection of F_2_s involving selection for bacterial late blight, blast, plant height, and visual grain yield was conducted under direct-seeded conditions. The best plants with enhanced plant type, disease resistance, grain type, and grain yield were carried forward in successive generations until the F_4_ generation. The plant-to-progeny testing of selected F_4_ plants was attempted in the F_5_ and F_6_ generation followed by an observational yield trial (OYT) and advanced yield trial (AYT) (Figure 1). The selection of disease resistance (blast and bacterial late blight) and grain quality was conducted in AYTs. The disease screening protocol followed was as described by Sandhu et al. (2018). The advanced breeding lines were analyzed for grain quality parameters at the IRRI GQNC (Grain Quality and Nutrition Center) laboratory facility. The detailed information on the grain quality testing is presented in Yadav et al. (2020). In the F_5_ and F_6_ generations, the progenies were tested in replicated yield trials with a plot size of 1.6 m^2^, whereas the plot sizes in OYT and AYT were 3.2 and 4.8 m^2^, respectively. In each generation, the plants were grown maintaining 20 cm (hill to hill) × 20 cm (row to row) distance. Detailed information on soil characteristics of upland fields, land preparation, field management, and phenotypic screening was provided by Sandhu et al. (2019b).

### Selection of Traits, Donors, and QTL for the Genomics-Assisted Introgression Program

Seedling emergence and establishment traits selected for the development of direct-seeded adapted rice varieties included early uniform emergence (germination rate, uniformity, and the percentage of seedling emergence; Dixit et al., 2015) and early vegetative vigor (higher relative growth rate and biomass accumulation, Sandhu et al., 2015). The abiotic stress tolerance traits included anaerobic germination (germination under flooding conditions; Ismail et al., 2009; Angaji et al., 2010) and drought tolerance (Vikram et al., 2011; Mishra et al., 2013). Root traits improving nutrient uptake, adaptability, and grain yield under direct-seeded cultivation conditions included nodal root, root hair length, root hair density, and root plasticity (Sandhu et al., 2015, 2016). The biotic stress tolerance/resistance traits included resistance to blast (Fjellstrom et al., 2004; Koide et al., 2011; Shikari et al., 2013), brown plant hopper (Sun et al., 2005; Jairin et al., 2007), gall midges (Nair et al., 1996), and bacterial blight (Song et al., 1997; Chu et al., 2006; Perumalsamy et al., 2010; Ullah et al., 2012). The other traits included lodging resistance (Dixit et al., 2015) and nematode tolerance (Galeng-Lawilao et al., 2018).

During the last 10 years of research at IRRI, the donors and QTL associated with these traits of interest were identified. At the IRRI, QTL for anaerobic germination (*qAG*_9.1_, Angaji et al., 2010), early uniform emergence (*qEMM*_1, 1_*, qEMM*_11.1_; Dixit et al., 2015), early vigor (*qEVV*_9.1_; Sandhu et al., 2015), higher root length density (*qNR*_4.1_, *qNR*_5.1_, *qRHD*_1.1_*, qRHD*_5.1_*, qRHD*_8.1_; Sandhu et al., 2015), facilitating higher N, P, and K uptake (*qN*_5.1_) under variable anaerobicaerobic soil conditions, lodging resistance (*qLDG*_4.1_, Dixit et al., 2015), and grain yield under dry direct-seeded conditions (*qGY*_1.1_*, qGY*_6.1_*, qGY*_10.1_; Sandhu et al., 2015) were identified. In addition to the above-mentioned QTL, those for increased yield under drought conditions (*qDTY*_1.1_*, qDTY*_2.1_*, qDTY*_3.1_*, qDTY*_12.1_) (Vikram et al., 2011; Mishra et al., 2013) have been identified. Previously reported genes for biotic stress resistance were used in the genomics-assisted introgression program. Detailed information on the donors and QTL selected and used in the present genomics-assisted introgression study is presented in Table 2.

**Table 2:** Detailed information on the donors and QTL selected and used in the genomics-assisted introgression study.

**S. No**.	**Trait**	**Donor**	**Population**	***QTL/genes***	**Interval markers**	**References**
1	Anaerobic germination	IR 93312-30-101-20-13-66-6	Donor: Khao Hlan On, Recipient: IR64	*qAG_9.1_*	RM8303-RM5526	Angaji et al., 2010
2	Early uniform emergence	IR 91648-B-32-B	Moroberekan/Swarna	*qEUE_1.1_, qEUE_11.1_*	id1100085-id11001535	Dixit et al., 2015
3	Early vigor, nodal root	IR 94226-B-177-B	Aus276/MTU1010	*qNR_5.1_* (Donor*: Aus276*)*, qEVV_9.1_* (*Donor: Aus276*)*, qRHD_1.1_* (*Donor: Aus276*)	Flanking Markers: id5000759-id5001182, ud9000737-id9002704, id1005271-id1006691; Peak Markers: id5001182, ud9000737, id1005271	Sandhu et al., 2015
4	Drought tolerance	IR 74371-46-1-1	IR 74371-46-1-1/2^*^Sabitri	*qDTY_12.1_, qDTY_2.3_, qDTY_3.2_*	*qDTY_12.1_*: RM28166-RM28199, *qDTY_2.3_*: RM3212-RM250, *qDTY_3.2_*: RM22-RM545	Mishra et al., 2013
5	Drought tolerance	IR 96322-34-223	Donors: N22, Apo; N22/Swarna, Apo/2^*^Swarna	*qDTY_3.1_, qDTY_1.1_, qDTY_2.1_*	*qDTY_3.1_*: RM416; *qDTY_1.1_*: RM11943-RM12091; *qDTY_2.1_*: RM324	Vikram et al., 2011
6	Grain yield	IR 94225-B-82-B	Aus276/IR64	*qGYDS_1.1_, qGYDS_6.1_*(Donor: *Aus276*)*, qGYDS_8.1_*(Donor: *IR64*)*, qGYDS_9.1_, qGYDS_10.1_*(Donor: *Aus276), qNR_4.1_* (Donor: *IR64*)	Flanking Markers: id6010515-id6015531, id8000536-id8000845, id10005369-id10006378, id4001205-id4002844; Peak Markers: id6015531, id8003773/ud8001270, id10006378, id4001205	Sandhu et al., 2015
7	Blast resistance	WHD-1S-75-1-127	Recipient: IR49830-7-1-2-2, BC_6_F_5_ population	*Pi9*	RM19814-RM3	Koide et al., 2011
8	Blast resistance	Tadukan	Germplasm screening, used as resistant check	*Pita2*	RM7102, RM155	Fjellstrom et al., 2004; Shikari et al., 2013
9	BPH	Rathu Hennati	Recipient: KDML105 (BC_3_F_2_)	*BPH3*	RM589-RM588 (Short arm of chromosome 6)	Jairin et al., 2007
10	BPH	Rathu Hennati	Recipient: 02428 (F_2_ population)	*BPH17*	RM8213-RM5953 (Short arm of chromosome 4)	Sun et al., 2005
11	Gall midge	Abhaya	F_1_, F_2_ and F_3_ populations from cross of BG380-2 and Gurmatia with Abhaya	*Gm4*	Chromosome 4: E20-E20570 (Susceptible), E20-E20583 (Resistance)	Nair et al., 1996
12	Bacterial blight	IRBB60	F_2_ populations of IRBB60/ADT43 and IRBB60/ASD16	*Xa4+xa5+xa13+Xa21*	*Xa4*: STS, MP1&MP2, *xa5*: FM-F, FM-R; RM122 (F & R); RG556; *xa13* gene: (CAPS)RG136, RP7 and ST12; *Xa21:* pTA248	Song et al., 1997; Chu et al., 2006; Perumalsamy et al., 2010; Ullah et al., 2012

### Genomics-Assisted Introgression Program

To combine anaerobic germination, blast resistance, brown plant hopper resistance, bacterial blight resistance, gall midge resistance, grain yield under direct-seeded cultivation conditions, early vigor, nodal roots, and early and uniform emergence in the background of high-yielding irrigated rice cultivar IR09N538 (IRRI 132/PR 30138-35-2//IR04N114) with preferable grain type, a complex crossing program began in the 2014 DS with 12 donors, including donors for biotic and abiotic stress tolerance and DSR-adapted traits. Based on the synchronization of flowering and availability of a true F_1_ generation with desired trait/QTL combinations, different cross combinations were attempted to achieve success in the breeding program. A detailed schematic representation of the pre-breeding steps and the genomics-assisted breeding strategy followed for the introgression of QTL/genes to develop a multi-stress-tolerant, direct-seeded, adapted superior rice genotype suitable for DDSR cultivation is presented in Figure 1.

### Genotyping

In the genomics-assisted introgression program, the F_1_s from biparental and multiparental crosses were tested in each season to check for true F_1_s using rice microsatellite (SSR; simple sequence repeats) markers. The SSR markers were identified in the previously reported QTL associated with traits, such as early uniform germination, early vegetative vigor, nodal roots, root hair length, root hair density, grain yield under direct-seeded conditions, and drought stress tolerance. A total of 1420 SSR markers in the QTL regions were used for parental polymorphism. Eighty-one SSR markers, one indel, and eight gene-specific polymorphic markers were used to screen the F_1_s and the progenies. A detailed description of the markers used to check the introgressed region is presented in Table 3.

**Table 3:** Detailed information on the SSR/InDel/gene-specific markers used in the genomics-assisted introgression study.

**QTL/gene**	**Chromosome location**	**QTL Span (bp)**	**Peak marker position (bp)**	**Markers**
*qEMM_1.1_*	1	172923-1172387	172923	RM10012, RM10043, RM10076, RM6887, RM495
*qEMM_11.1_*	11	2725632-6879889	2725632	RM26076, RM26092, RM26279, RM26321
*qEVV_9.1_*	9	12251875-12254087	12252981	RM24351
*qNR_4.1_*	4	2524875-7349119	2524875	RM5414, RM16424, RM16428, RM8213, RM6487, RM1305, RM16556, RM16686, RM16672
*qNR_5.1_*	5	1103913-1956488	1956488	RM17885, RM3345, RM5796
*qRHD_1.1_*	1	6989731-8959532	8959532	RM5989, RM8098, RM6784, RM259, RM1032, RM10701
*qRHD_5.1_*	5	8101358-16491275	8101358	RM18166, RM18149, RM18173, RM18354, RM18360
*qRHD_8.1_*	8	1835532-2921777	2921777	RM22306, RM1376
*qGY_1.1_*	1	39463573-43348002	43204288	RM11943, RM6333, RM431, RM12147, RM5310, RM122281, RM12092, RM12289, RM12276
*qGY_6.1_*	6	20701496-28259604	28259604	RM20493, RM20535, RM20632, RM20633
*qGY_10.1_*	10	18663448-20607217	20607217	RM25457, RM25745, RM1108, RM25895
*qDTY_1.1_*	1	37846103-38888469	38367286	RM431, RM11943, RM12023, RM12146, RM12233
*qDTY_2.1_*	2	9600368-12020819	11211992	RM324, RM3549, RM12868, RM5791, RM12987, RM12995
*qDTY_3.1_*	3	30718826-32500578	31609702	RM520, RM416, RM16030
*qDTY_12.1_*	12	14153465-18225086	16189275.5	RM28048, RM28099, RM28166, RM28199, RM511, Indel8
*qAG_9.1_*	9	12251875-12254087	12252981	DFR_F2, DFR_R2, DFR_LB2
*GM4*	8	5583984-5587025	5585504.5	Gene specific marker (GM4_LRR-del_F, GM4_LRR-del_R)
*Xa4*	11	27673251-27673372	27673311.5	Gene specific marker (Xa4_MP1, Xa4_MP2)
*xa5*	5	437010-443270	440140	Gene specific marker (xa5_F2_Sus, xa5_F2_Res, xa5_R2)
*xA13*	8	26725898-26728795	26727346.5	Gene specific marker (xa13F, xa13R)
*XA21*	11	21274459-21277323	21275891	Gene specific marker (xa21F, xa21R)
*Pi9*	6	10382004-10390596	10386300	Gene specific marker (Pi9-659T_F, Pi9-659T_R), Pi9-1477G_F, Pi9-1477G_R, M492, M493
*Pita2*	12	10606359-10612157	10609258	M535, M536, YL155, YL87, YL153, YL154
*BPH3*	6	1380931-1611442	1476905	RM586, RM589, RM7639, RM19311, RM190
*BPH17*	4	4418222-9713776	7065999	RM8213, RM6487, RM16430, RM16431, RM16556, RM16567

Fresh, young leaves from the 15-day-old rice seedlings were collected from each F_1_ plant and the respective parents. Genomic DNA was extracted from F_1_s using a modified CTAB protocol (Murray and Thompson, 1980). The DNA from the phenotypically selected plants from generations F_2_ onwards was used for further studies. The amplification of genomic region using PCR (polymerase chain reaction), separation of bands on agarose, and 6–8% (v/v) PAGE (polyacrylamide gel electrophoresis (CBS scientific model MGV-202-33); depending on the SSR, indel, and gene-specific marker product size was performed. After staining with SYBR Safe™, the separated genomic fragments were visualized under a UV trans-illuminator (AlphaImager™ System). The associations of the polymorphic SSRs, indel, and gene-specific markers with the introgressed genomic regions were exploited for the accurate detection of the F_1_s and the progenies. Because there are many introgressed genomic regions/genes and many are not fine mapped, a sequential two-stage genotyping approach was used to make the genotyping labor and cost effective. The first stage selection was performed on phenotypically selected plants using the peak markers associated with the introgressed genomic region. The second-stage selection was performed on the selected plants harboring the selected donor allele using flanking and all other markers underlying the QTL regions.

### Phenotyping

To combine traits of true F_1_s to increase rice adaptation under direct-seeded cultivation conditions, –early uniform emergence, early vigor, high-nutrient uptake through improved root traits, tolerance to drought, and grain yield under conditions together with resistance to biotic stresses were selected using trait-linked markers. The number of F_1_s developed per cross per season is listed in Supplementary Table 1. At each generation, phenotypic plant selection was conducted initially based on plant type, duration, plant height, number of tillers, grain type, and visual yield, and then selected plants were evaluated genotypically with the trait associated markers. A single plant selection strategy was followed from the F_2_ to F_6_ generation for the presence of QTL/gene combinations together with phenotypic selection for yield to reject plants showing negative interactions. A plot size of 8 m^2^ was used for the screening of each breeding line at the F_4_, F_5_, and F_6_ generation. At each advancing generation step, the plants were grown maintaining 20 cm (hill to hill) × 20 cm (row to row) distance. Field management was conducted following the procedure described by Sandhu et al. (2019b). The data on plant height (cm) and grain yield of single plant selection (g) and plot yield (kg ha^−1^) were collected. Plant height was measured from the root-shoot junction to the tip of the uppermost panicle on the main tiller. After harvesting at maturity, the grains were first dried to 14% moisture, and then weighed to record GY (g, kg ha^−1^).

### Statistical Analysis

The agronomic data collected from all the conventional and GAB experiments were analyzed using statistical tools and software. The experimental means and standard error of difference of the progeny testing experiments were calculated using IRRI *PBTools v1.4*. The least significant difference (LSD) at *P* = 1 and 5% levels of significance were used to compare the means of test entries and to estimate the significant variations existing between parents and the breeding lines for the particular trait of interest. An ANOVA (analysis of variance) was estimated using following mixed linear model:

(1) $$\begin{array}{r} Yijk=u+Gi+Rj+BK\left(Rj\right)+eijk \end{array}$$

where μ, Gi, Rj, BK(Rj), and eijk are the overall mean, effect of ith breeding line, effect of jth replicate, block effect of the jth replicate, and error, respectively. While estimating the entry mean, the genotypes and replication block effects were kept as fixed and random, respectively.

### Mean Comparison of Different Parental Classes

The hypothesis regarding mean differences among the breeding lines developed involving different numbers of parental lines under direct-seeded cultivation conditions was performed using the following linear model in SAS v9.2 (SAS Institute Inc. 2009).

(2) $$\begin{array}{r} Y_{\text{ijkl}}=\mu +rk+b\left(r\right)kl+qi+g\left(q\right)ij+eijkl \end{array}$$

where μ, rk, b(r)kl, qi, g(q)ij, and eijkl symbolize the population mean, effect of the kth replicate, effect of the lth block within the kth replicate, effect of the ith parental class, effect of the jth breeding line nested within the ith parental class, and the error, respectively (Knapp, 2001). The effects of parental class and the breeding lines within the parental class were considered as fixed effects, and the replicates and blocks effects within replicates were set as random.

## Results

### Conventional Breeding Program

A total of 184 crosses involving 137 biparent crosses, 21 triparent crosses, 16 quadraparent, 5 pentaparent, and 5 hexaparent crosses were attempted and evaluated from the F_2_ generation for advanced yield trials. A highly stringent phenotypic selection based on plant type, duration, plant height, number of tillers, grain type, and visual yield across generations was made. The stringency of selection can be estimated from the number of plants/breeding lines selected across generation advancement, as represented in Supplementary Table 2.

The crosses involving three or more parents showed grain yield improvement over the biparental lines and the upland adapted check varieties (Table 4). The high mean grain performance of the parental class involving more than two parents in the F_4_ generation was consistent in the F_5_ and F_6_ generations and also in observational and advanced yield trials across seasons. The highest average grain yield was observed in the hexaparent class across generations and seasons (Table 4). The average grain yield of the biparent class ranged from 3,517 to 4,589 kg ha^−1^ in the WS (wet season) and from 5,010 to 5,796 kg ha^−1^ in the DS across generations. The parental class involving three, four, and five parents showed average grain yields ranging from 4,434 to 5,904 kg ha^−1^, 4,532 to 6,002 kg ha^−1^, and 4,566 to 6,124 kg ha^−1^, respectively, across generations. The grain yield of the hexaparent class ranged from 4,680 to 5,603 kg ha^−1^ in the WS and from 5,505 to 6,292 kg ha^−1^ in the DS across generations. Across season and generation advancement, the grain yield advantage of the biparent class ranged from 100 to 540 kg ha^−1^, triparent class from 35 to 1,008 kg ha^−1^, and quadraparent class from 352 to 1,095 kg ha^−1^ over the check varieties. The parental classes involving five and six parents consistently outperformed the check varieties in terms of average grain yield advantage (Table 4). The multi-parent lines had significantly higher yield (2,072–6,569 kg ha^−1^) than the bi-parental lines (1,493–6,326 kg ha^−1^) at the F_6_ generation in the DS.

**Table 4:** Mean comparison of QTL classes for grain yield (kg ha^−1^) from the F_4_ to F_6_ generations in observational and advanced yield trials under direct-seeded cultivation conditions.

**Parental class**	**2016 WS**	**2016 WS**	**2016 WS**	**2017DS**	**2017WS**	**2016WS**	**2017DS**	**2017WS**	**2016WS**	**2017DS**	**2017WS**
	**F_**4**_**	**F_**5**_**	**F_**6**_**	**F_**6**_**	**F_**6**_**	**OYT**	**OYT**	**OYT**	**AYT**	**AYT**	**AYT**
A	3,962 a	4,598 a	4,397 ab	5,796 a	4,346 a	4,436 a	5,474 a	3,517 a	4,082 b	5,010 a	4,270 a
B	4,434 b	4,775 a	4,961 bc	5,817 b	4,521 ab	4,503 a	5,904 b	4,443 c	4,630 c	5,194 ab	4,543 ab
C	–	–	4,711 bc	5,889 b	5,154 b	4,532 a	6,002 b	4,556 c	–	5,211 ab	4,862 b
D	4,912 c	5,069 b	–	6,109 b	5,323 bc	4,566 a	6,124 bc	4,743 c	–	5,299 ab	4,973 bc
E	–	5,203 b	5,511 c	6,029 b	5,562 c	5,003 b	6,292 c	4,680 c	–	5,505 c	5,288 c
F	3,862 a	4,282 a	4,119 a	5,537 a	4,059 a	4,106 a	5,352 ab	4,090 b	3,542 a	4,827 a	4,508 ab
Trial mean	4,292	5,088	4,740	5,868	4,820	4,516	5,852	4,335	4,085	5,174	4,740
*F*-value	20.25	9.73	7.43	10.8	2.82	31.6	8.82	43.87	8.59	12.82	8.16
*P*-value	<0.0001	0.0005	0.0005	0.0013	0.0802	<0.0001	<0.0001	<0.0001	0.0004	<0.0001	<0.0001

*A, biparent cross; B, triparent cross; C, quadraparent cross; D, pentaparent cross; E, hexaparent cross; F, check varieties; WS, wet season; DS, dry season; OYT, observational yield trial; AYT, advanced yield trial; means followed by same letter within a column indicate the particular QTL classes were not significantly different from each other*.

The breeding lines developed involving more than two parents could ensure 0.3–1.5 kg ha^−1^ improvement in grain yield over the check varieties and 0.1–1.3 kg ha^−1^ grain yield improvement over the breeding lines involving two parents. DTF of the different classes were comparable to the check varieties, except in some seasons where 1 or 2 d earliness was observed (Table 5). Most of the classes showed comparable or slightly higher PHT than did the check varieties (Table 5).

**Table 5:** Mean comparison of QTL classes for DTF (d) and PHT (cm) from the F_4_ to F_6_ generations in observational and advanced yield trials under direct-seeded cultivation conditions.

	**DTF**									**PHT**								
**Parental class**	**2016 WS**	**2017 DS**	**2017WS**	**2016 WS**	**2017 DS**	**2017 WS**	**2016 WS**	**2017DS**	**2017 WS**	**2016 WS**	**2017DS**	**2017WS**	**2016 WS**	**2017 DS**	**2017 WS**	**2016 WS**	**2017 DS**	**2017 WS**
	**F_**6**_**	**F_**6**_**	**F_**6**_**	**OYT**	**OYT**	**OYT**	**AYT**	**AYT**	**AYT**	**F_**6**_**	**F_**6**_**	**F_**6**_**	**OYT**	**OYT**	**OYT**	**AYT**	**AYT**	**AYT**
A	77 b	96 b	76 b	76 c	81 c	72.16 c	75 a	79 ab	71.33 c	101 a	96 a	113 a	101 a	100 a	112 a	103 a	103 b	115 a
B	78 cd	96 b	76 b	75 b	81 c	71.12 b	77 b	78 a	70.93 b	104 ab	98 ab	114 a	110 c	102 ab	114 ab	103 ab	94 a	115 a
C	77 bc	95 a	77 b	76 b	79 b	71.49 b	–	79 ab	71.62 c	99 a	97 ab	118 b	112 c	98 a	114 ab	–	97 a	114 a
D	–	95 a	76 b	75 b	79 b	71.68 b	–	78 a	70.45 b	–	100 b	114 a	110 c	107 ab	116 b	–	105 b	115 ab
E	74 a	95 a	77 b	74 a	76 a	72.11 c	–	79 ab	68.60 a	113 c	100 b	116 b	102 b	114 b	115 b	–	112 c	118 b
F	78 d	95 a	74 a	80 d	80 c	70.10 a	77 b	80 b	74.05 d	106 b	100 b	116 b	103 ab	98 ab	115 ab	107 b	103 b	117 ab
Trial mean	77	95	76	76	80	71.51	76	79	71.30	105	99	115	106	102	114	104	103	116
*F*-value	7.34	6.45	6.52	251.5	19.4	13.26	18	2.2	72.88	11.54	6.53	12.66	25.99	1.54	12.98	3.51	89.79	3.35
*P*-value	0.0006	0.0105	0.006	<0.0001	<0.0001	<0.0001	<0.0001	0.095	<0.0001	<0.0001	0.010	0.000	<0.0001	0.201	<0.0001	0.0346	<0.0001	0.0139

*A, biparent cross; B, triparent cross; C, quadraparent cross; D, pentaparent cross; E, hexaparent cross; F, check varieties; WS, wet season; DS, dry season; OYT, observational yield trial; AYT, advanced yield trial; means followed by same letter within a column indicate the particular QTL classes are not significantly different from each other*.

### Genomics-Assisted Breeding Program

**Figure 2:**
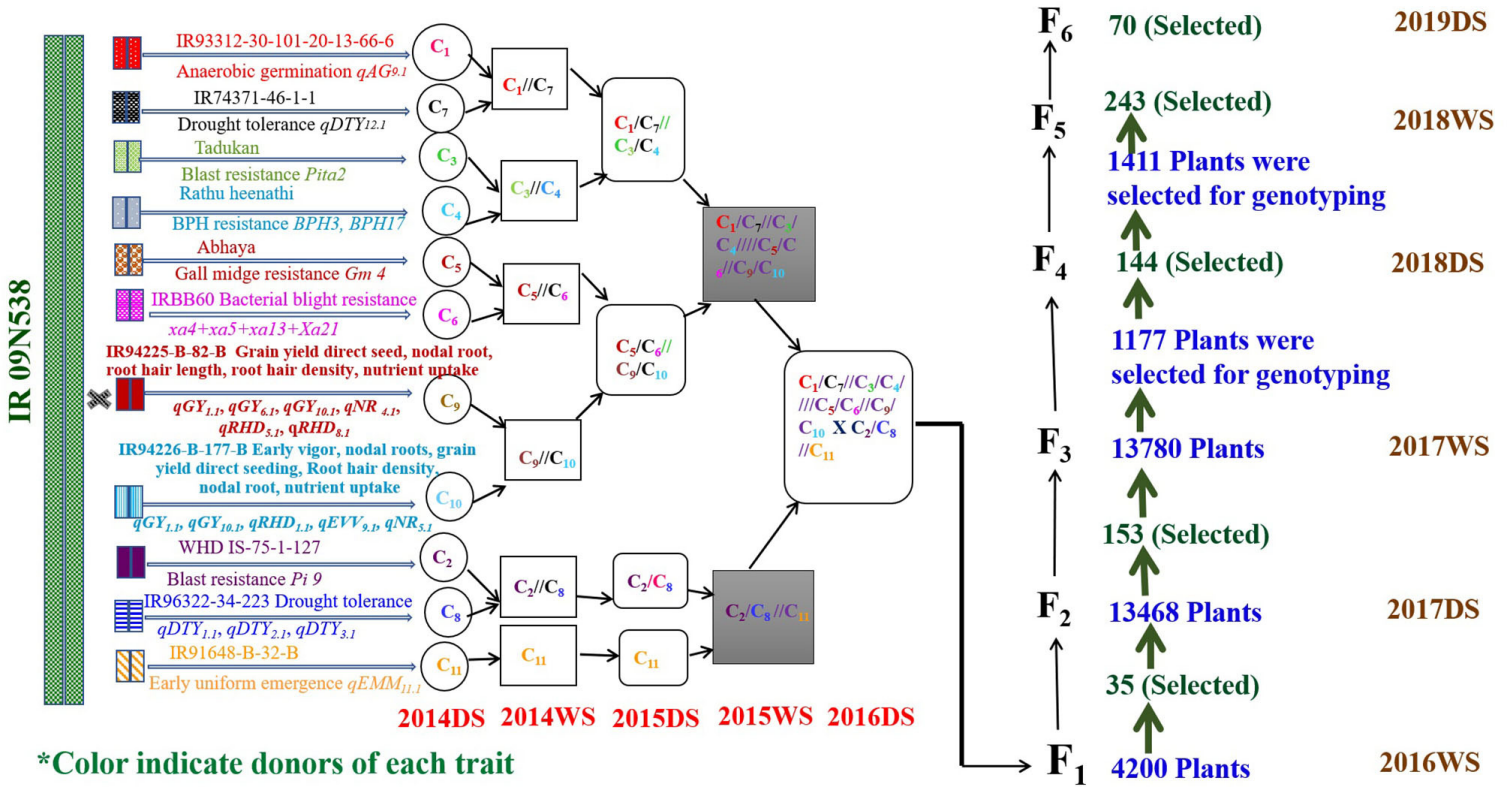
Detailed schematic representation of crossing strategy followed for the introgression of QTL/genes from 12 donors in the background of IR09N538 and the number of plants selected across successive generations. #C: represents the cross.

The GAB program involving 12 donors and IR09N538 as the recipient was initiated in the 2014 DS. The final multiparent F_1_s were generated in the 2016DS Figure 2. A single plant selection strategy was followed across generations. The multiparental 4,200 F_1_s were grown in an F_1_ nursery at IRRI under puddled transplantation conditions in the 2016WS. The number of QTL/genes varied from two to 15 and grain yield ranged from 1.0 to 58.8 g/plant. Thirty-five F_2_s derived F_1_s were selected based on different QTL/gene combinations. The number of QTL/genes in the selected plants ranged from 8 to 15 and the grain yield varied from 8.0 to 58.8 g/plant. An F_2_ population of approximately 40,000 plants, F_3_ population of 13,780 plants, F_4_ population of 144 breeding lines, F_5_ population of 243 breeding lines, and F_6_ population of 70 breeding lines were raised and screened under DDSR cultivation conditions. A total of 13,468 F_2_ plants of required plant type, duration, plant height, number of tillers, grain type, and visual yield were screened genotypically with the trait-associated markers, and a total of 153 F_2_ derived F_3_ plants were selected. The number of QTL/genes in the selected plants varied from 3 to 12, plant height from 80 to 130 cm, and grain yield from 5.9 to 68 g/plant. The 1,177 F_3_ derived F_4_ plants and 1,411 F_4_ derived F_5_ plants were selected and advanced by combining phenotypic genotypic selection in the 2017WS and the 2018DS, respectively. More than 30 genomics-assisted derived F_5_ breeding lines with different combinations of QTL/genes yielded more than check varieties MTU1010, IRRI 155, UPLRi7, and the recipient parent, IR09N538. F_4_ derived F_5_ breeding lines with 223 different combinations of QTL/genes were evaluated in the 2018WS. Significant variability in grain yield, ranging from 1,026 to 5,956 kg ha^−1^, was observed (data not shown). After genotypic selection at the F_5_ level, a total of 427 single plants were selected phenotypically, and then 70 plants to progeny were carried forward to the F_6_ generation. The grain yield variability in the selected F_6_ breeding lines varied from 3,293 to 6,719 kg ha^−1^, plant height from 65 to 115 cm, and days to 50% flowering from 77 to 104 d (data not shown). The QTL/genes in selected F_6_ breeding lines varied from 6 to 12, and a total of 55 different combinations of QTL/genes with acceptable phenotype and grain characteristics were selected.

### Comparison of Conventional and Genomics-Assisted Derived Breeding Lines

The analysis of the differences in mean grain yield of genomics-assisted derived breeding lines was significantly higher than that of breeding lines derived from conventional breeding programs Table 6. The grain yield of GAB lines derived from multi-parent crosses ranged from 3,293 to 6,719 kg ha^−1^; however, the grain yield of multi-parent lines from conventional breeding ranged from 2,072 to 6,569 kg ha^−1^ at the F_6_ generation in the DS. The GAB lines IR 129477-2815-41-4-7-1, IR 129477-3343-500-36-5-1, IR 129477-4139-439-1-1-2, and IR 129477-1629-14-1-2-2 with eight, nine, nine, and 11 QTL/genes, respectively, showed grain yield of more than 5,500 kg ha^−1^ in the WS at the F_5_ generation advancement stage and more than 6,000 kg ha^−1^ in the DS at the F_6_ generation Table 7. The breeding lines IR 115844-B-B-475-1-2, IR 107891-B-B-1253-1-1, IR 115845-B-B-296-2-1, and IR 115846-B-B-197-1-1 from four different crosses of the conventional breeding program involving five/six parents yielded more than 4,500 kg ha^−1^ in the WS at the F_5_ generation advancement stage and more than 5,500 kg ha^−1^ in the DS at the F_6_ generation (Table 7). The genomics-assisted derived breeding lines showed better yield performance (5,100–7,004 kg ha^−1^) when tested at Varanasi, India, under observational yield trials (Table 7). The breeding lines from the conventional breeding program showed delayed flowering compared to the genomics-assisted breeding lines, except the lines derived from the cross Dular (acc. 32561)/IRRI 148///IRRI 154/UPLRi 7//IR87707-446-B-B-B/Kali Aus (Table 7). The genomics-assisted derived breeding lines were more dwarf than the breeding lines developed from the conventional breeding program (Table 7). Further, the breeding lines developed from the crosses (NSIC Rc 222/IR87707-446-B-B-B)/IR87707-446-B-B-B/IRRI148//Vandana/Kali Aus, and Dular (acc. 32561)/IRRI 148///IRRI 154/UPLRi 7//IR87707-446-B-B-B/Kali Aus showed better performance across generation advancement followed by (Sahbhagi dhan/UPLRi 7)/(PSBRc 82/Kali Aus)//(Vandana/IRRI148) and (Sahbhagi dhan/IR87707-446-B-B)/(PSBRc 82/IRRI148)//(Kali Aus/Kalinga 3) (Table 7).

**Table 6:** Analysis of the differences for grain yield (kg ha^−1^) at the F_6_ generation in the dry season between the categories of breeding lines (with a confidence interval of 95%) developed following genomic assisted breeding and conventional breeding approaches.

**Breeding program**	**Category**	**Mean**	**Standard error**	**Lower bound** ** (95%)**	**Upper bound** ** (95%)**
Genomics-assisted breeding	Genomics-assisted breeding lines	5,050 a	83.23	4,886.51	5,213.09
Conventional breeding	Dular (acc. 32561)/IRRI 148///IRRI 154/UPLRi 7//IR87707-446-B-B-B/Kali Aus	4,649 b	35.08	4,580.12	4,717.78
	(NSIC Rc 222/IR87707-446-B-B-B)/IR87707-446-B-B-B/IRRI 148//Vandana/Kali Aus	4,574 b	45.88	4,484.19	4,664.22
	(Sahbhagi dhan/UPLRi 7)/(PSBRc 82/Kali Aus)//(Vandana/IRRI148)	4,563 b	52.76	4,459.68	4,666.71
	(Sahbhagi dhan/IR87707-446-B-B)/(PSBRc 82/IRRI 148)//(Kali Aus/Kalinga 3)	4,529 b	85.76	4,360.71	4,697.22
	(Swarna/IR87707-446-B-B)/(PSBRc 82/IRRI 148)//(Vandana/IRRI 148)	4,473 bc	177.07	4,126.02	4,820.83
	(NSIC Rc 222/WAB 880-1-27-9-2-P1-HB)/(IR87707-446-B-B/Dular)//(Dular/Kalinga 3)	4,110 c	177.07	3,762.65	4,457.46

*Means followed by same letter within a column indicate the particular classes are not significantly different from each other*.

**Table 7:** Mean comparison of grain yield (kg ha^−1^), DTF (days), and PHT (cm) of breeding lines developed under the genomics-assisted breeding program and conventional breeding program.

**Breeding program**	**Designation**	**QTL/parentage**	**No of QTL/gene**	**GY**			**DTF**	**PHT**	**GY**	**DTF**	**PHT**
				**DS**	**WS**	**DS**	**DS**	**DS**	**WS**	**WS**	**WS**
				${\text{F}}_{4}^{*}$	**F_**5**_**	**F_**6**_**	**F_**6**_**	**F_**6**_**	**OYT**	**OYT**	**OYT**
Genomics-assisted breeding	IR 129477-1629-14-1-4-2	*Xa4* + *xa5* + *Xa21* + *BPH3* + *Pi9* + *Pita* + *qAG_9.1_*+ *qDTY_3.1_*+ *qNR_5.1_*+ *qRHD_1.1_*+ *qEMM_1.1_*	11	21.8	5,956	5,768	97	89	5,510	95	101
	IR 129477-1629-14-1-2-2	*Xa4 + xa5 + Xa21 + BPH3 + Pi9 + Pita + qAG_9.1_ + qDTY_3.1_ + qNR_5.1_ + qRHD_1.1_ + qEMM_1.1_*	11	35.4	5,665	6,068	84	91	–	–	–
	IR 129477-4026-249-15-1-2	*Xa4* + *Xa21* + *BPH3* + *GM4* + *qAG_9.1_* + *qDTY_3.1_* + *qDTY_12.1_* + *qRHD_1.1_* + *qRHD_5.1_*+ *qEMM_11.1_*	10	47.4	5,739	5,741	86	101	6,338	92	82
	IR 129477-1629-210-4-4-4	*xa5 + Xa21 + BPH3 + Pita + qAG_9.1_ + qDTY_2.1_ + qDTY_3.1_ + qNR_5.1_ + qRHD_1.1_ + qEMM_1.1_*	10	25.6	5,944	5,874	89	85	5,477	93	94
	IR 129477-902-121-10-1-1	*Xa4* + *BPH3* + *GM4* + *Pita* + *qAG_9.1_* + *qDTY_3.1_* + *qGY_6.1_*+ *qGY_10.1_* + *qNR_5.1_* + *qNR_4.1_*	10	30.4	5,852	5,743	84	95	5,860	94	79
	IR 129477-4139-439-1-1-2	*Xa4*+ *xa5*+ *Xa21*+ *Pi9* + *Pita* + *qAG_9.1_*+ *qDTY_3.1_*+ *qDTY_12.1_*+ *qEMM_11.1_*	9	30.4	5,870	6,118	77	97	7,358	85	109
	IR 129477-3343-500-36-5-1	*Xa4* + *xa5* + *xa13* + *GM4* + *Pita*+ *qDTY_3.1_*+ *qAG_9.1_*+ *qRHD_1.1_*+ *qEMM_11.1_*	9	25.8	5,714	6,169	87	86	6,999	83	96
	IR 129477-2815-41-4-7-1	*Xa4 + BPH3 + Pita + Pita2 + qAG_9.1_ + qDTY_3.1_ + qGY_6.1_ + qGY_10.1_*	8	33.0	5,940	6,719	84	87	–	–	–
	IR 129477-4197-209-2-2-2	*Xa4 + xa5 + BPH3 + Pita + Pita2 + qAG_9.1_ + qDTY_3.1_ + qNR_5.1_*	8	49.4	5,699	5,871	100	87	5,076	96	98
	IR 129477-2064-233-1-1-3	*Xa4 + GM4 + Pita + qAG_9.1_ + qDTY_3.1_ + qNR_5.1_ + qRHD_1.1_*	7	35.6	5,860	6,461	85	79	–	–	–
	IR 129477-709-375-3-5-7	*GM4* + *Pita*+ *qAG_9.1_*+ *qDTY_3.1_*+ *qDTY_12.1_*+ *qGY_6.1_*+ *qNR_5.1_*	7	46.2	5,626	5,880	85	88	6,683	98	100
	IR09N538		–	14.7	3,717	4,437	89	89	–	–	–
	UPLRi7	–	–	16.1	3,856	5,014	92	92	–	–	–
	IRRI 155	–	–	13.6	4,015	4,959	82	99	–	–	–
	MTU1010	–	–	14.4	4,031	4,992	83	82	4,165	90	91
	Trial mean			28.7	3,206	4,376	91	88	4,696	91	94
	LSD			–	1,376	828	3	10	1,130	5	8
Conventional breeding	IR 107891-B-B-1060-1-1	Dular (acc. 32561)/IRRI 148///IRRI 154/UPLRi 7//IR87707-446-B-B-B/Kali Aus	–	4,664	4,438	6,048	76	115	6,072	74	118
	IR 107891-B-B-601-2-1	Dular (acc. 32561)/IRRI 148///IRRI 154/UPLRi 7//IR87707-446-B-B-B/Kali Aus	–	5,025	4,579	6,375	77	114	6,281	75	111
	IR 107891-B-B-1253-1-1	Dular (acc. 32561)/IRRI 148///IRRI 154/UPLRi 7//IR87707-446-B-B-B/Kali Aus	–	5,221	4,718	6,569	76	110	6,338	72	119
	IR 107891-B-B-958-3-1	Dular (acc. 32561)/IRRI 148///IRRI 154/UPLRi 7//IR87707-446-B-B-B/Kali Aus	–	5,073	4,644	5,938	76	107	5,578	73	117
	IR 115843-B-B-543-1-1	(NSIC Rc 222/WAB 880-1-27-9-2-P1-HB)/(IR87707-446-B-B/Dular)//(Dular/Kalinga 3)	–	4,933	4,759	5,749	93	95	–	–	–
	IR 115844-B-B-475-1-2	(NSIC Rc 222/IR87707-446-B-B-B)/IR87707-446-B-B-B/IRRI148//Vandana/Kali Aus	–	5,478	5,390	6,323	93	108	6,240	73	115
	IR 115844-B-342-1-1-1	(NSIC Rc 222/IR87707-446-B-B-B)/IR87707-446-B-B-B/IRRI148//Vandana/Kali Aus	–	5,330	4,855	6,032	95	111	6,061	72	120
	IR 115844-B-B-281-1-2	(NSIC Rc 222/IR87707-446-B-B-B)/IR87707-446-B-B-B/IRRI148//Vandana/Kali Aus	–	5,263	4,712	5,889	94	112	6,345	72	122
	IR 115844-B-B-638-1-1	(NSIC Rc 222/IR87707-446-B-B-B)/IR87707-446-B-B-B/IRRI148//Vandana/Kali Aus	–	5,490	4,945	5,735	94	107	6,203	72	117
	IR 115845-B-222-1-1-2	(Sahbhagi dhan/UPLRi 7)/(PSBRc 82/Kali Aus)//(Vandana/IRRI148)	–	4,713	4,845	6,051	94	102	5,934	72	113
	IR 115846-B-B-197-1-1	(Sahbhagi dhan/IR87707-446-B-B)/(PSBRc 82/IRRI148)//(Kali Aus/Kalinga 3)	–	5,088	5,573	5,602	94	95	5,950	67	120
	IR 115847-B-B-6-1-2	(Swarna/IR87707-446-B-B)/(PSBRc 82/IRRI148)//(Vandana/IRRI148)	–	4,778	4,572	5,994	96	98	–	–	–
	IRRI 155			4,300	3,978	5,497	90	105	4,387	110	74
	UPLRi7			4,533	4,008	5,667	98	111	–	–	–
	Trial Mean			4,211	4,133	4,483	95	98	4,687	102	75
	LSD			502	448	199	4	11	546	7	4

* *Single plant yield of selected plant (g)*.

*WS, wet season; DS, dry season; OYT, observational yield trial; DTF, days to 50% flowering; PHT, plant height*.

### Selection of Promising Lines/Donors

**Figure 3:**
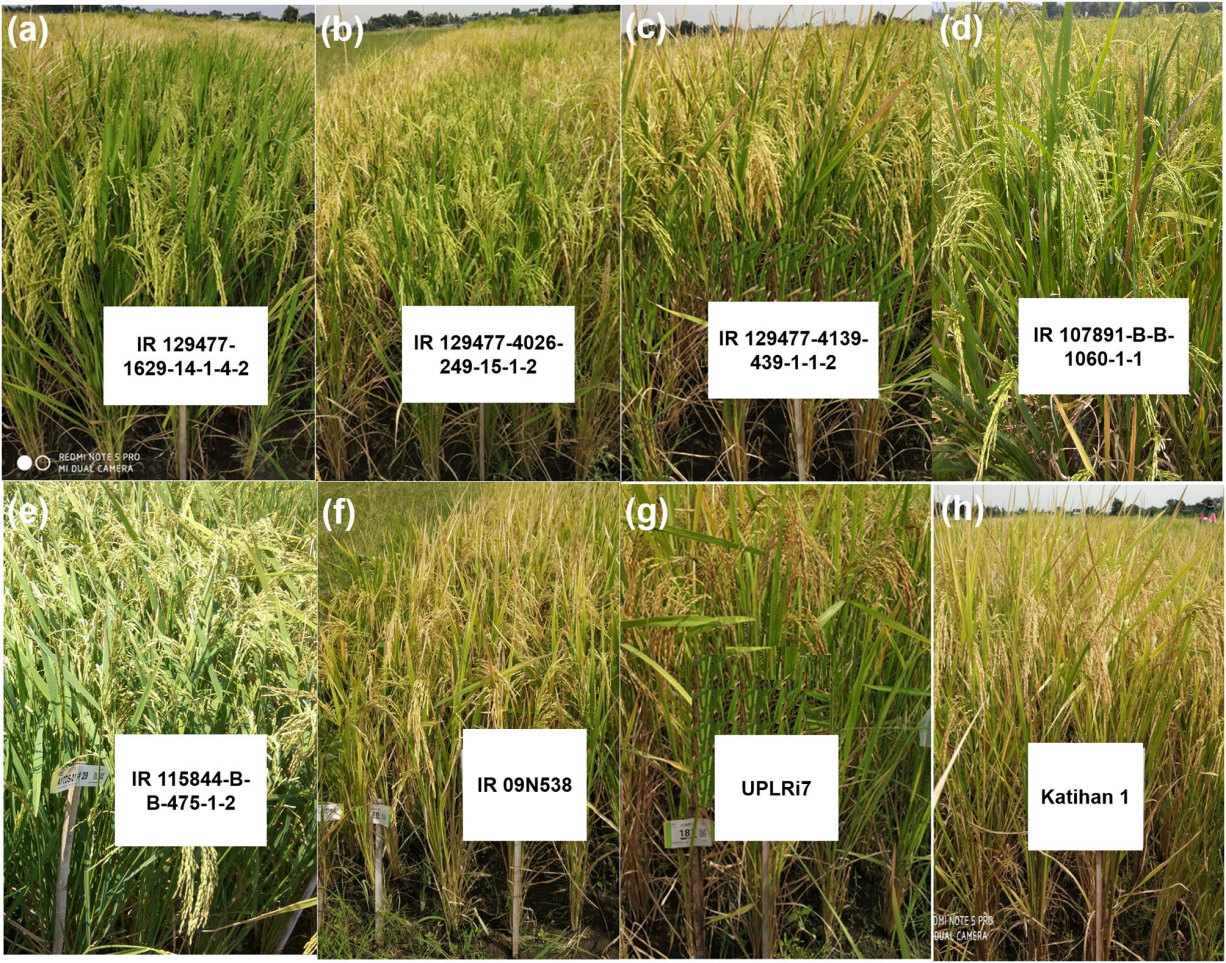
Selected promising breeding lines from **(a–c)** genomics-assisted breeding program **(d,e)**, conventional breeding program, and **(f)** recipient variety used in genomics-assisted breeding program **(g,h)** for upland adapted check varieties.

The breeding lines carrying multiple QTL/genes from GAB programs and multiparent lines from conventional breeding programs performed well in terms of grain yield and adaptability under DDSR and also exhibited phenotypically high levels of tolerance/resistance to abiotic and biotic stresses with desired grain quality characteristics. A total of 11 promising lines carrying 7–11 QTL/genes for various DDSR adaptable traits, as well as biotic stresses tolerance/resistance (Table 7, Figures 3a–c), and eight promising lines from the multiparental conventional breeding program were selected (Table 8, Figures 3d,e) for further evaluation in multiple environments for varietal release. The recipient variety IR 09N538 is presented in Figure 3f and the upland adapted check varieties, UPLRi7 and Katihan 1 are presented in Figures 3g,h, respectively. It is interesting to note that the genomics-assisted breeding lines with a combination of DDSR adaptable traits and abiotic stress tolerance/resistance QTL/genes showed better yield advantages, *viz*. IR 129477-2815-41-4-7-1 (*Xa4* + *BPH3* + *Pita* + *Pita2* + *qAG*_9.1_ + *qDTY*_3.1_ + *qGY*_6.1_ + *qGY*_10.1_) showed ~33% and IR 129477-1629-14-1-2-2 (*Xa4* + *xa5* + *Xa21* + *BPH3* + *Pita* + *qAG*_9.1_+ *Pi9* + *qDTY*_3.1_ + *qNR*_5.1_ + *qRHD*_1.1_ + *qEMM*_1.1_) showed 22% yield improvement over the best performing check variety. These lines were 6–7 d earlier than the other lines. The breeding lines IR 115844-B-B-281-1-2 and IR 115844-B-342-1-1-1 from five parent cross (NSIC Rc 222/IR87707-446-B-B-B)/IR87707-446-B-B-B/IRRI148//Vandana/Kali Aus yielded more than 7,000 kg ha^−1^ under direct-seeded cultivation conditions in an advanced yield trial (Table 8). Most of the selected breeding lines possessed less chalkiness, medium amylose content, and intermediate GT content (Table 8). Lines with chalkiness >10% and amylose content <10% were not carried forward in the breeding program. The promising breeding lines from both the conventional and GAB programs can be further tested for adaptability and yield stability by conducting multi-location trials in their targeted environments. These pyramided lines may serve as novel donors for the development of lines with multiple abiotic and biotic stress tolerance/resistance traits.

**Table 8:** Performance of selected promising multi-parent breeding lines over check varieties from a conventional breeding program in advanced yield trials at IRRI, Philippines.

**Designation**	**DDSR**			**NS**			**RS**			**DDSR**			**Grain quality**				**Biotic stress**		
	**DTF**	**PHT**	**GY**	**DTF**	**PHT**	**GY**	**DTF**	**PHT**	**GY**	**DTF**	**PHT**	**GY**	**Clk**	**Amy**	**GT**	**% HR**	**Bl**	**PXO61**	**PXO 86**
	**DS**	**DS**	**DS**	**DS**	**DS**	**DS**	**DS**	**DS**	**DS**	**WS**	**WS**	**WS**							
IR 107891-B-B-1060-1-1^#^	76	95	6,284	79	124	5,605	76	83	2,397	68	125	6,105	6.2	25.8	I	59.4	1	3	7
IR 107891-B-B-958-3-1^#^	78	83	5,892	79	122	5,574	77	80	1,850	66	124	6,443	6.2	23.6	I	55.3	0	1	5
IR 107891-B-B-1253-1-1^#^	76	92	6,153	80	114	6,567	75	84	3,043	68	124	6,414	15.8	25.2	I	45.0	0	3	7
IR 115844-B-342-1-1-1^†^	77	98	7,108	84	115	6,755	80	74	2,973	91^*^	119^*^	5,343^*^	1.6	25.2	L	51.1	0	3	7
IR 115844-B-B-281-1-2^†^	80	100	7,556	82	111	6,939	79	83	3,541	87^*^	119^*^	5,188^*^	4.4	24.8	L	43.4	0	3	5
IR 115844-B-B-638-1-1^†^	79	90	6,168	83	113	5,811	81	76	2,721	–	–	–	5.7	24.8	L	33.1	0	3	7
IR 115844-B-B-561-1-1^†^	79	99	6,044	79	105	5,496	78	75	2,519	–	–	–	9.5	22.9	I	40.2	1	3	7
IR 115845-B-222-1-1-2^γ^	77	99	6,694	79	111	6,134	79	84	3,109	89^*^	119^*^	5,021^*^	5.6	23.3	I/L	50.2	0	3	7
IR 115845-B-438-1-1-1^γ^	93	97	6,662	79	108	6,546	80	78	2,827	87^*^	116^*^	4,347^*^	1.7	25.2	L	44.8	0	3	7
Katihan 1	77	99	5,567	77	113	5,369	75	77	2,100	70/87^*^	129/123^*^	5,883/4,562^*^	1.1	25.0	I	55.8	–	–	–
Sahod Ulan 6	82	104	5,828	88	104	6,243	87	75	1,741	81/91^*^	114/108^*^	2,810/4,446^*^	0.5	24.5	L	58.9	–	–	–
Vandana	74	93	4,777	76	104	6,030	73	77	1,579	69/81^*^	120/114^*^	4,870/3,854^*^	1.4	23.1	L	54.5	–	–	–
IR 74371-70-1-1	75	92	5,812	75	103	4,888	71	78	2,264	69	114	3,824	7.0	22.3	L	54.2	–	–	–
MTU 1010	76	92	5,447	81	108	5,268	78	76	2,212	68	109	3,724	12.4	22.4	HI	55.8	–	–	–
Trial Mean	78	95	5,244	81	107	5,455	79	75	2,243	68/89^*^	125/109^*^	4,460/4,652^*^	–	–	–	–	–	–	–
LSD	8	7	677	1	5	541	1	5	428	1/1^*^	2/5^*^	1,088/449^*^	–	–	–	–	–	–	–

# *Dular (acc. 32561)/IRRI 148///IRRI 154/UPLRi 7//IR87707-446-B-B-B/Kali Aus*.

† *NSIC Rc 222/IR87707-446-B-B-B)/IR87707-446-B-B-B/IRRI148//Vandana/Kali Aus*.

γ (Sahbhagi dhan/UPLRi 7)/(PSBRc 82/Kali Aus)//(Vandana/IRRI148), Katihan 1, IR 74371-70-1-1: IR 55419-04/Way rarem, Sahod Ulan 6: WS 91/Abhaya//IR 43070-UBN 511-2-1-1-1, IR 74371-70-1-1: IR 55419-4^*^2/Way rarem.

* *Lines tested in different years but in the WS season*.

*DS, dry season; WS, wet season; DDSR, direct-seeded cultivation conditions; RS, reproductive stage drought stress; NS, transplanted non-stress control; DTF, days to 50% flowering (d); PHT, plant height (cm); GY, grain yield (kg ha^−1^); Clk, chalkiness; Amy, amylose content; GT, Gelatinization temperature; %HR, percent head rice recovery; BL, blast screening; PXO61, PXO86, screening for bacterial blight, blast, and bacterial blight score: 0: highly resistant, 1–2: resistant, 3–4: moderately resistant, 5–6: moderately susceptible, and 7–8: susceptible*.

## Discussion

To meet the gaps between rice crop yields and global rice consumption, rice breeders need to continuously release new rice varieties with better yield potential, adaptability under DDSR, acceptable grain quality traits, high nutrient-use efficiency, and resistance to various biotic/abiotic stresses (Leng et al., 2017). The longstanding idea behind using an insecticide mixture to broaden the insect resistance spectrum laid the foundation of gene pyramiding (Ye and Smith, 2008). The nature of donors/traits selected for introgression, number of genes transferred, distance between the introgressed genes and flanking markers, and the number of plants selected with different gene combinations across each breeding generation, are critical for an effective QTL/genes pyramiding program.

To the best of our knowledge, the present study is the first to report the pyramiding of 7–11 QTL/genes associated with both traits, providing better adaptability and biotic stress tolerance/resistance under DSSR, exploiting genomics-assisted foreground selection. Previous studies reported the marker-assisted pyramiding of QTL for either tolerance to abiotic stress traits, such as drought and submergence (Swamy et al., 2013; Septiningsih et al., 2015; Shamsudin et al., 2016; Dixit et al., 2017; Sandhu et al., 2019a), or resistance to blast (Singh et al., 2013; Fukuoka et al., 2015), bacterial blight (Suh et al., 2013; Pradhan et al., 2015; Das et al., 2018), brown plant hoppers (Wang et al., 2015; Jena et al., 2017), and gall midges (Divya et al., 2015) in rice.

Various high-yielding rice varieties from the conventional breeding program involving phenotypic selection (Sandhu and Kumar, 2017) and genomics-assisted selection (Sandhu et al., 2019b) have been released. However, labor and time use, linkage drag, and low efficiency are some of the major barriers impeding conventional breeding (Prohens, 2011). Advances in molecular mapping, precise phenotyping, novel marker development, and multi-parent population development have paved the way for genomics-assisted breeding. These advances have gradually shifted the focus of traditional plant breeding from phenotype-based selection to genotype-based selection (Xu and Crouch, 2008). The GAB strategies involve backcrossing or introgression of QTL/genes, enrichment of multiple favorable alleles, and selection for introgressed traits/QTL/genes using trait-linked markers (Hospital et al., 1992; Eathington et al., 2007; Gupta et al., 2010).

A GAB program combining 25 QTL/genes and a conventional breeding program involving multi-parent cross-governing better adaptation of rice to direct-seeded cultivation conditions was undertaken at IRRI. The final step of the introgression program was to fix the targeted QTL/genes into a homozygous state across successive generations. Generally, recombinant inbred lines (RILs) and double haploid (DH) production techniques are used for the development of homozygous lines. We did not succeed in combining all 25 QTL/genes in one breeding line because: (i) some with large introgressed regions were lost during successive recombination events, (ii) some QTL/genes combination altered the flowering cycle and led to the failure of planned crossing strategy in successive seasons because of non-synchronization of flowering, (iii) some of the complex F_1_s crosses with possible negative interactions among the introgressed QTL/genes were rejected and not utilized for further crossing, and (iv) some of the QTL that were in a heterozygous state were lost in succeeding generations. We succeeded in pyramiding 15 QTL/genes in the F_2_/F_3_ generation in the background of IR09N538 but some QTL/genes, which were in a heterozygous state, were lost in succeeding generations. In this regard, DH production has been reported to provide satisfactory results in becoming fully homozygous in a very short time (Dunwell, 2010; Mishra and Rao, 2016), which was not used in the present study. Additionally, genetic engineering (GE) also aids the precise transfer of genes of interest to crop plants (Lemaux, 2008) to generate crops with desired trait/s. Although DH and GE are precise, universal, and fast methods to transfer the desired gene/s into different crop plants (Nicholl, 2008), it will not replace GAB, but it will definitely add to the efficiency of rice crop improvement. Some QTL/genes combination that showed negative interactions leading to poor grain yield and adaptability under DDSR with unacceptable plant and grain type were rejected in successive generations. Finally, the pyramided lines possessing 7–11 QTL/genes under homozygous conditions were selected, indicating the existence of positive interactions among different biotic and abiotic stress-related QTL/genes.

The DDSR lines developed through conventional and GAB strategies were evaluated at an advanced stage under dry direct-seeding conditions. The increased yield of breeding lines developed under conventional breeding programs involving multi-parents performed over the bi-parental lines across generations could result from the accumulation of alleles for traits that increase rice adaptability to direct-seeded situations (Huang et al., 2012). It can be predicted that, with the use of a greater number of parents, there will be greater identification of the complex traits (Huang et al., 2012), a greater number of recombinations, and greater chances of accumulation of favorable alleles. Nevertheless, multi-parent crosses require more time and higher costs for the development of the population, but increased genetic variations may provide opportunities to improve grain yield and adaptability under DDSR. In the present study, we had succeeded in breaking the unfavorable linkages, elimination of the inferior plants with poor plant and grain type during the selection process. The better grain yield performance of GAB-derived multi-parental breeding lines compared to the breeding lines developed from conventional breeding programs could be the result of precise marker-based selection that helps reduce undesirable linkage drags. GAB aids in obtaining the desirable QTL/genes combination without any unwanted genes, minimizing the linkage drag around the target QTL/genes. GAB has been proven effective for transferring QTL/genes from pyramided lines into improved varieties and new plants (Magar et al., 2014).

A stepwise hybridization and selection strategy in the GAB program led to the selection of the most suitable plants with desired QTL/gene combinations. Arbelaez et al. (2019) reported that an average of 800 F_2_ individuals plants were required to obtain 50 lines with the desired genotype fixed at two loci. Compared to the F_2_ generation, the frequency of desired homozygotes for two linked genomic loci will decrease nearly three times in the F_6_ generation (Arbelaez et al., 2019). Keeping the above points in view, a large population size of 40,000 (F_2_) and 13,780 (F_3_) plants capturing the hidden genetic variations were maintained in the early generations to select appropriate plants possessing positive interactions among targeted QTL/gene combinations, with desired plant type, grain type, better grain yield, and adaptability under DDSR. The increased yield of the GAB-derived lines from multiparent crosses has been achieved, keeping the plant height and days to 50% flowering, similar to check varieties, also indicates yield increase as a result of accumulation of favorable alleles, increasing rice adaptability to direct-seeded situations caused by introgression of genes/QTL for such traits. Notably, stepwise phenotypic and genotypic selection in the early generations has reduced the genotypic efforts, finally reducing the genotyping cost. The effectiveness of the above-mentioned strategy targeting the pyramiding of various drought QTL has been successfully demonstrated in various genetic backgrounds by Kumar et al. (2018).

Most of the GAB-improved lines developed in the present study carry the *xa5* gene in combination with *xa13* and *Xa21* genes. Based on previous reports, *xa5* (Jiang et al., 2006; Huang et al., 2016), *xa13* (Ogawa et al., 1987; Chu et al., 2006), and *Xa21* (Huang et al., 1997; Cao et al., 2005) genes are considered as the most effective R genes, providing broader levels of resistance, and have been widely used for introgression programs against BLB in most rice growing countries in Asia. The selected promising lines carrying either the *Pita, Pita2*, or *Pi9* gene alone showed stable resistance to blast disease. Similarly, Qu et al. (2006) reported that the *Pi9* gene can provide broader and durable resistance against rice blast disease. Additionally, the selected breeding lines possessed a genomic region providing resistance to gall midge, tolerance to reproductive stage drought stress, adaptability, and improved yield under DDSR. The selected improved breeding lines carrying the genomic region contributing to biotic stress resistance will further be evaluated phenotypically. The better performance of breeding lines under transplanted control non-stress and reproductive stage drought stress conditions (Table 8), in addition to DDSR, indicated their phenotypic plasticity. Enhanced seedling establishment, improved and stable yield, and phenotypic plasticity of the root system have been reported to result in improved adaptability of selected promising lines across variable growing conditions (Sandhu et al., 2016, 2017). Grain quality parameters play a crucial role in varietal release and wider acceptance of the released varieties (Rani et al., 2006). Thus, our field selection criteria also involved selection for grain type across each successive generation. The breeding lines from the conventional and GAB programs were selected considering the preferences of various countries in South and South East Asia. The developed breeding lines possessing medium amylose content are suitable for India and Nepal, those with higher amylose content for Bangladesh, and those with lower amylose content for South East Asia.

## Conclusions

The study showed GAB was a better strategy for improving yield and adaptation of rice under DDSR utilizing multiple QTL/genes compared to conventional breeding. A higher number of parents and more recombination events resulted in higher grain yield advantage compared to the classical bi-parental population. These findings are vital for researchers working on multi-parental populations, because this will remarkably reduce the efforts of developing large numbers of bi-parental populations. The rigorous selection from the F_2_ generation onwards resulted in the development of promising breeding lines carrying 7–11 QTL/genes for different traits. Promising GAB lines carrying multiple QTL/genes under homozygous conditions with preferable grain type and quality traits can address the constraints faced by farmers caused by both biotic and abiotic stresses simultaneously under DDSR in addition to the labor-water shortage. The GAB-derived breeding lines and multi-parent conventional breeding lines could further be recommended for varietal release after multi-location evaluation in national and provincial coordinated trials in different countries. These breeding lines may serve as a novel genetic resource that could aid breeders and molecular biologists to further conduct genetic and interaction studies for a wide range of breeder-relevant parameters.

## Data Availability Statement

The original contributions presented in the study are included in the article/Supplementary Material, further inquiries can be directed to the corresponding author/s.

## Author Contributions

NS conducted the experiment, analysis, data interpretation, and drafting of the manuscript. SY assisted in conducting experiments. MCa assisted in genotyping. MCr performed management of field experiments. AK conceived the study and was involved in critical revision and final approval of the manuscript. All authors thoroughly discussed the results.

## Conflict of Interest

The authors declare that the research was conducted in the absence of any commercial or financial relationships that could be construed as a potential conflict of interest.
