# Supplementary material for: Developing Climate-Resilient, Direct-Seeded, Adapted Multiple-Stress-Tolerant Rice Applying Genomics-Assisted Breeding
Source: Front Plant Sci. 2021 Apr 15;12:637488. doi: 10.3389/fpls.2021.637488 (PMC8082028; doi:10.3389/fpls.2021.637488)
Supplement: Supplementary file 1 [file Table_1.DOCX]

**Developing climate-resilient, direct-seeded adapted multiple-stress tolerant rice applying genomic assisted breeding**

Nitika Sandhu^1,2^, Shailesh Yadav^1^, Margaret Catolos^1^, Ma. Teresa Sta Cruz^1^, Arvind Kumar^1,3*^

^1^Rice Breeding Platform, International Rice Research Institute, DAPO Box 7777, Metro Manila, Philippines

^2^Punjab Agricultural University, Ludhiana, Punjab, India

^3^IRRI South Asia Regional Centre (ISARC), Varanasi, Uttar Pradesh, 221106, India

* Correspondence: Arvind Kumar ([a.kumar@irri.org](mailto:a.kumar@irri.org))

**Table S1** The detailed information on the number of F_1_s produces and selected per cross per season from 2012 to 2016

| 2012DS-2014DS | | 2014DS | 2014WS | | 2015DS | | 2015WS | | 2016DS | | 2016WS | |
| --- | --- | --- | --- | --- | --- | --- | --- | --- | --- | --- | --- | --- |
| Recipient | **Donor** | **No. of F_1s_ produced** | **No. of F_1s_ produced** | **No. of plants selected** | **No. of F_1s_ produced** | **No. of plants selected** | **No. of F_1s_ produced** | **No. of plants selected** | **No. of F_1s_ produced** | **No. of plants selected** | **No. of F_1s_ produced** | **No. of plants selected** |
| IR09N538 | IR93312-30-101-20-13-66-6 | 100 | 315 | 60 | 1,806 | 194 | 5,953 | 1,059 | 15,224 | 4,150 | 37,474 | 4,200 |
|  | IR74371-46-1-1 | 100 |  |  |  |  |  |  |  |  |  |  |
|  | Tadukan | 100 | 200 | 64 |  |  |  |  |  |  |  |  |
|  | Rathu hennathi | 100 |  |  |  |  |  |  |  |  |  |  |
|  | Abhaya | 100 | 155 | 32 | 2,257 | 221 |  |  |  |  |  |  |
|  | IRBB60 | 100 |  |  |  |  |  |  |  |  |  |  |
|  | IR 94225-B-82-B | 100 | 200 | 76 |  |  |  |  |  |  |  |  |
|  | IR 94226-B-177-B | 100 |  |  |  |  |  |  |  |  |  |  |
|  | WHD-1S-75-1-1-127 | - | 100 | 36 | 2,684 | 204 | 3,833 | 674 |  |  |  |  |
|  | IR 96322-34-223 | - | 100 | 108 |  |  |  |  |  |  |  |  |
|  | IR 91648-B-32-B | - | - | - | 507 | 98 |  |  |  |  |  |  |

**Table S2** The detailed information on the number of plants/breeding lines selected across successive generations

| Cross type | Parentage | 2010  DS | 2011  WS | 2012  DS | 2012  WS | 2013  DS | 2013  WS | 2014  DS | 2014  WS | 2015  DS | 2015  WS | 2016  DS | 2016  WS | 2017  DS | 2017  WS | 2018  DS | 2018  WS |
| --- | --- | --- | --- | --- | --- | --- | --- | --- | --- | --- | --- | --- | --- | --- | --- | --- | --- |
| Biparent | BR 28/Arang |  |  |  |  |  |  |  |  | 50 (F_1_) | 60 (F_2_) | 79 (F_3_) | 17 (F_4_) | 21 (F_5_) | 50 (F_6_) | 5 (OYT) | 1 (AYT) |
|  | BR 28/Banda |  |  |  |  |  |  |  |  | 37 (F_1_) | 59 (F_2_) | 80 (F_3_) | 41 (F_4_) | 73 (F_5_) | 224 (F_6_) | 40 (OYT) | 9 (AYT) |
|  | BR 28/Bea Balok Loas |  |  |  |  |  |  |  |  | 44 (F_1_) | 51 (F_2_) | 38 (F_3_) | 22 (F_4_) | 28 (F_5_) | 50 (F_6_) | 4 (OYT) | - |
|  | BR 29/Beaq Pendjalin |  |  |  |  |  |  |  |  | 100 (F_1_) | 45 (F_2_) | 41 (F_3_) | 21 (F_4_) | 39 (F_5_) | 79 (F_6_) | 14 (OYT) | 2 (AYT) |
|  | BR 29/Benong 130 |  |  |  |  |  |  |  |  | 49 (F_1_) | 52 (F_2_) | 37 (F_3_) | 9 (F_4_) | 13 (F_5_) | 20 (F_6_) | 3 (OYT) | - |
|  | BR 29/Boder |  |  |  |  |  |  |  |  | 44 (F_1_) | 56 (F_2_) | 38 (F_3_) | 15 (F_4_) | 28 (F_5_) | 60 (F_6_) | 5 (OYT) | - |
|  | IR09N538/IR 96322-34-223-B |  |  |  |  |  |  |  |  | 121 (F_1_) | 58 (F_2_) | 66 (F_3_) | 13 (F_4_) | 14 (F_5_) | 40 (F_6_) | 4 (OYT) | - |
|  | IR09N538/WHD IS-75-1-127 |  |  |  |  |  |  |  |  | 75 (F_1_) | 59 (F_2_) | 68 (F_3_) | 25 (F_4_) | 32 (F_5_) | 65 (F_6_) | 7 (OYT) | 3 (AYT) |
|  | IRRI 154/IR 94226-B-177 |  |  |  |  |  |  |  |  | 100 (F_1_) | 46 (F_2_) | 22 (F_3_) | 7 (F_4_) | 10 (F_5_) | 15 (F_6_) | 3 (OYT) | - |
|  | IRRI 154/IR 96322-34-223-B |  |  |  |  |  |  |  |  | 59 (F_1_) | 30 (F_2_) | 40 (F_3_) | 17 (F_4_) | 27 (F_5_) | 44 (F_6_) | 5 (OYT) | 1 (AYT) |
|  | IRRI 154/WHD IS-75-1-127 |  |  |  |  |  |  |  |  | 45 (F_1_) | 60 (F_2_) | 63 (F_3_) | 31 (F_4_) | 51 (F_5_) | 128 (F_6_) | 15 (OYT) | 4 (AYT) |
|  | MTU 1010/IR04N114 |  |  |  |  |  |  |  |  | 78 (F_1_) | 33 (F_2_) | 21 (F_3_) | 11 (F_4_) | 17 (F_5_) | 25 (F_6_) | 1 (OYT) | 1 (AYT) |
|  | HHZ 8-SAL 6-SAL 3-Y2/Bunga Mehu |  |  |  |  |  |  |  |  | 64(F_1_) | 48 (F_2_) | 118 (F_3_) | 76 (F_4_) | 119 (F_5_) | 282 (F_6_) | 35 (OYT) | 16 (AYT) |
|  | HHZ 8-SAL 6-SAL 3-Y2/Cicih Gedu |  |  |  |  |  |  |  |  | 71 (F_1_) | 55 (F_2_) | 58 (F_3_) | 28 (F_4_) | 50 (F_5_) | 140 (F_6_) | 18 (OYT) | 6 (AYT) |
|  | HHZ 8-SAL 6-SAL 3-Y2/Kam |  |  |  |  |  |  |  | 59 (F_1_) | 242 (F_2_) | 56 (F_3_) | 27 (F_4_) | 10 (F_5_) | 20 (F_6_) | - | - | - |
|  | HHZ 8-SAL 6-SAL 3-Y2/Khilak |  |  |  |  |  |  |  | 100 (F_1_) | 179 (F_2_) | 69 (F_3_) | 51 (F_4_) | 17 (F_5_) | 32 (F_6_) | 1 (OYT) | - | - |
|  | IR 106542/IRRI 123 |  |  |  |  |  |  |  | 69 (F_1_) | 34 (F_2_) | 75 (F_3_) | 71 (F_4_) | 20 (F_5_) | 30 (F_6_) | 2 (OYT) | - | - |
|  | IR 91648-B-114-B-1-B/HHZ 8-SAL 6-SAL 3-Y2 |  |  |  |  |  |  |  | 61 (F_1_) | 24 (F_2_) | 75 (F_3_) | 73 (F_4_) | 22 (F_5_) | 44 (F_6_) | 4 (OYT) | 2 (AYT) | - |
|  | IR 91648-B-114-B-1-B/PR 30245-10-414 |  |  |  |  |  |  |  | 45 (F_1_) | 29 (F_2_) | 76 (F_3_) | 68 (F_4_) | 30 (F_5_) | 60 (F_6_) | 4 (OYT) | 3 (AYT) | 1 (AYT) |
|  | IR 91648-B-114-B-1-B/PR 35805-B-9-2-3-2-3 |  |  |  |  |  |  |  | 45(F_1_) | 52 (F_2_) | 105 (F_3_) | 99 (F_4_) | 27 (F_5_) | 54 (F_6_) | 1 (OYT) | - | - |
|  | IR 91648-B-117-B-1-1/HHZ 8-SAL 6-SAL 3-Y2 |  |  |  |  |  |  |  | 57 (F_1_) | 34 (F_2_) | 78 (F_3_) | 73 (F_4_) | 22 (F_5_) | 42 (F_6_) | - | - | - |
|  | IR 91648-B-117-B-1-1/PR 37139-3-1-3-1-2-1 |  |  |  |  |  |  |  | 51 (F_1_) | 28 (F_2_) | 75 (F_3_) | 73 (F_4_) | 35 (F_5_) | 66 (F_6_) | 3 (OYT) | - | - |
|  | IR 91648-B-117-B-1-1/PR 37866-1B-1-4 |  |  |  |  |  |  |  | 50 (F_1_) | 40 (F_2_) | 70 (F_3_) | 60 (F_4_) | 32 (F_5_) | 64 (F_6_) | 7 (OYT) | - | - |
|  | IR 91648-B-117-B-1-1/UPL RI 7 |  |  |  |  |  |  |  | 50(F_1_) | 27 (F_2_) | 70 (F_3_) | 42 (F_4_) | 13 (F_5_) | 26 (F_6_) | 1 (OYT) | - | - |
|  | IR 91648-B-117-B-1-1/WAB 880-1-27-9-2-P1-HB |  |  |  |  |  |  |  | 46 (F_1_) | 49 (F_2_) | 28 (F_3_) | 18 (F_4_) | 4 (F_5_) | 6 (F_6_) | 2 (OYT) | - | - |
|  | IR 91648-B-1-B-3-1/HHZ 8-SAL 6-SAL 3-Y2 |  |  |  |  |  |  |  | 66 (F_1_) | 48 (F_2_) | 69 (F_3_) | 38 (F_4_) | 24 (F_5_) | 48 (F_6_) | 4 (OYT) | 1 (AYT) | - |
|  | IR 91648-B-1-B-3-1/PR 37942-3B-5-3-2 |  |  |  |  |  |  |  | 49 (F_1_) | 36 (F_2_) | 69 (F_3_) | 50 (F_4_) | 38 (F_5_) | 74 (F_6_) | 4 (OYT) | 1 (AYT) | 1 (AYT) |
|  | IR 91648-B-1-B-3-1/PR 37951-3B-37-1-2 |  |  |  |  |  |  |  | 61 (F_1_) | 30 (F_2_) | 69 (F_3_) | 48 (F_4_) | 41 (F_5_) | 80 (F_6_) | 4 (OYT) | - | - |
|  | IR 91648-B-285-B-3-1/TME 80518 |  |  |  |  |  |  |  | 70 (F_1_) | 34 (F_2_) | 69 (F_3_) | 62 (F_4_) | 14 (F_5_) | 28 (F_6_) | - | - | - |
|  | IR 91648-B-296-B-2-1/HHZ 8-SAL 6-SAL 3-Y2 |  |  |  |  |  |  |  | 50 (F_1_) | 42 (F_2_) | 69 (F_3_) | 63 (F_4_) | 16 (F_5_) | 28 (F_6_) | - | - | - |
|  | IR06N119/Kam |  |  |  |  |  |  |  | 50 (F_1_) | 84 (F_2_) | 71 (F_3_) | 28 (F_4_) | 7 (F_5_) | 14 (F_6_) | - | - | - |
|  | IR08L181/Banda |  |  |  |  |  |  |  | 50 (F_1_) | 168 (F_2_) | 72 (F_3_) | 40 (F_4_) | 14 (F_5_) | 28 (F_6_) | 1 (OYT) | - | - |
|  | IR08L181/Tjempo Krembung |  |  |  |  |  |  |  | 37 (F_1_) | 189 (F_2_) | 76 (F_3_) | 64 (F_4_) | 23 (F_5_) | 46 (F_6_) | 2 (OYT) | - | - |
|  | IR08L216/Gendjah Ratji |  |  |  |  |  |  |  | 44 (F_1_) | 212 (F_2_) | 70 (F_3_) | 9 (F_4_) | 5 (F_5_) | 10 (F_6_) | - | - | - |
|  | IR08L216/Ketan Gadjih Sungut |  |  |  |  |  |  |  | 59(F_1_) | 215 (F_2_) | 70 (F_3_) | 14 (F_4_) | 6 (F_5_) | 12 (F_6_) | - | - | - |
|  | IR09A228/Khilak |  |  |  |  |  |  |  | 65 (F_1_) | 122 (F_2_) | 66 (F_3_) | 54 (F_4_) | 28 (F_5_) | 56 (F_6_) | - | - | - |
|  | IR09L204/Ketan Merah |  |  |  |  |  |  |  | 29 (F_1_) | 113 (F_2_) | 69 (F_3_) | 47 (F_4_) | 10 (F_5_) | 20 (F_6_) | 1 (OYT) | - | - |
|  | IR10A134/Lakhon |  |  |  |  |  |  |  | 66 (F_1_) | 147 (F_2_) | 114 (F_3_) | 27 (F_4_) | 8 (F_5_) | 16 (F_6_) | 1 (OYT) | - | - |
|  | IRRI 123/Beaq Pendjalin |  |  |  |  |  |  |  | 100 (F_1_) | 79 (F_2_) | 59 (F_3_) | 59 (F_4_) | 16 (F_5_) | 32 (F_6_) | 1 (OYT) | - | - |
|  | IRRI163/Nogo Bele 2 |  |  |  |  |  |  |  | 84 (F_1_) | 125 (F_2_) | 69 (F_3_) | 40 (F_4_) | 9 (F_5_) | 18 (F_6_) | - | - | - |
|  | PR 30245-10-414/ Kam |  |  |  |  |  |  |  | 56 (F_1_) | 203 (F_2_) | 84(F_3_) | 79 (F_4_) | 52 (F_5_) | 104 (F_6_) | 8 (OYT) | - | - |
|  | PR 37139-3-1-3-1-2-1/Intok |  |  |  |  |  |  |  | 38 (F_1_) | 232 (F_2_) | 85 (F_3_) | 77 (F_4_) | 47 (F_5_) | 94 (F_6_) | 11 (OYT) | - | - |
|  | PR 37951-3B-37-1-2/Chao Med Nyay |  |  |  |  |  |  |  | 55 (F_1_) | 122 (F_2_) | 74 (F_3_) | 50 (F_4_) | 29 (F_5_) | 58 (F_6_) | 2 (OYT) | - | - |
|  | HHZ 5-DT20-DT2-DT1/IR09L204 |  |  |  |  |  |  | 59 (F_1_) | 56 (F_2_) | 28 (F_3_) | 50 (F_4_) | 51 (F_5_) | 76 (F_6_) | 12 (OYT) | 4 (AYT) | - | - |
|  | HHZ 5-DT20-DT2-DT1/IR09L336 |  |  |  |  |  |  | 100 (F_1_) | 52 (F_2_) | 42 (F_3_) | 31 (F_4_) | 30 (F_5_) | 40 (F_6_) | 10 (OYT) | 8 (AYT) | 2 (AYT) | - |
|  | HHZ 5-DT20-DT2-DT1/IR09L342 |  |  |  |  |  |  | 38 (F_1_) | 52 (F_2_) | 41 (F_3_) | 33 (F_4_) | 39 (F_5_) | 54 (F_6_) | 7 (OYT) | - | - | - |
|  | IR 74371-70-1-1/IR09L204 |  |  |  |  |  |  | 76 (F_1_) | 50 (F_2_) | 16 (F_3_) | 25 (F_4_) | 13 (F_5_) | 8 (F_6_) | 1 (OYT) | - | - | - |
|  | IR 74371-70-1-1/IR09L342 |  |  |  |  |  |  | 48 (F_1_) | 45 (F_2_) | 27 (F_3_) | 44 (F_4_) | 33 (F_5_) | 40 (F_6_) | 12 (OYT) | - | - | - |
|  | IR 74371-70-1-1/WAB 880-1-27-9-2-P1-HB |  |  |  |  |  |  | 55 (F_1_) | 36 (F_2_) | 22 (F_3_) | 21 (F_4_) | 16 (F_5_) | 12 (F_6_) | 4 (OYT) | 1 (AYT) | - | - |
|  | IR 87707-446-B-B-B/Kali Aus |  |  |  |  |  |  | 37 (F_1_) | 49 (F_2_) | 9 (F_3_) | 13 (F_4_) | 9 (F_5_) | 6 (F_6_) | 3 (OYT) | 1 (AYT) | - | - |
|  | IR 90265-B-551-1/IR 87707-445-B-B-B |  |  |  |  |  |  | 55 (F_1_) | 43 (F_2_) | 31(F_3_) | 31 (F_4_) | 27 (F_5_) | 22 (F_6_) | 6 (OYT) | 2 (AYT) | 1 (AYT) | - |
|  | IR 90265-B-551-1/IR 87707-446-B-B-B |  |  |  |  |  |  | 38 (F_1_) | 48 (F_2_) | 26 (F_3_) | 24 (F_4_) | 17 (F_5_) | 37 (F_6_) | 11 (OYT) | 1 (AYT) | - | - |
|  | IR BB 60 (IR 72920-1-44-4)/IR09L336 |  |  |  |  |  |  | 100 (F_1_) | 52 (F_2_) | 40 (F_3_) | 251 (F_4_) | 23 (F_5_) | 26 (F_6_) | 4 (OYT) | 2 (AYT) | - | - |
|  | IR BB 60 (IR 72920-1-44-4)/IR09L342 |  |  |  |  |  |  | 88 (F_1_) | 50 (F_2_) | 34 (F_3_) | 23 (F_4_) | 24 (F_5_) | 28 (F_6_) | 7 (OYT) | 1 (AYT) | - | - |
|  | IR09L179/B 1050 D-KN-1-1-1-1-3 |  |  |  |  |  |  | 58 (F_1_) | 150 (F_2_) | 34 (F_3_) | 31 (F_4_) | 11 (F_5_) | 29 (F_6_) | 5 (OYT) | - | - | - |
|  | IR09L179/Cicih gedu |  |  |  |  |  |  | 52 (F_1_) | 155 (F_2_) | 34 (F_3_) | 37 (F_4_) | 17 (F_5_) | 20 (F_6_) | 5 (OYT) | 1 (AYT) | - | - |
|  | IR09L204/Bunga Mehu |  |  |  |  |  |  | 64 (F_1_) | 101 (F_2_) | 70 (F_3_) | 21 (F_4_) | 16 (F_5_) | 22 (F_6_) | 6 (OYT) | 1 (AYT) | - | - |
|  | IR09L204/Cicih gedu |  |  |  |  |  |  | 66 (F_1_) | 30 (F_2_) | 42 (F_3_) | 54 (F_4_) | 47 (F_5_) | 72 (F_6_) | 14 (OYT) | 5 (AYT) | - | - |
|  | IR09L336/Cicih gedu |  |  |  |  |  |  | 47 (F_1_) | 118 (F_2_) | 50 (F_3_) | 28 (F_4_) | 27 (F_5_) | 28 (F_6_) | 6 (OYT) | 3 (AYT) | - | - |
|  | IR09L337/Bunga mehu |  |  |  |  |  |  | 40 (F_1_) | 141 (F_2_) | 52 (F_3_) | 63 (F_4_) | 51 (F_5_) | 76 (F_6_) | 38 (OYT) | 11 (AYT) | - | - |
|  | IR09L337/Cicih gedu |  |  |  |  |  |  | 55 (F_1_) | 104 (F_2_) | 45 (F_3_) | 14 (F_4_) | 12 (F_5_) | 10 (F_6_) | 2 (OYT) | - | - | - |
|  | IRRI 123/IR 87707-446-B-B-B |  |  |  |  |  |  | 78 (F_1_) | 51 (F_2_) | 55 (F_3_) | 69 (F_4_) | 64 (F_5_) | 60 (F_6_) | 5 (OYT) | 1 (AYT) | - | - |
|  | IRRI 123/MR 219 |  |  |  |  |  |  | 66 (F_1_) | 39 (F_2_) | 69(F_3_) | 31 (F_4_) | 40 (F_5_) | 62 (F_6_) | 9 (OYT) | - | - | - |
|  | IRRI 123/UPL RI 7 |  |  |  |  |  |  | 89 (F_1_) | 26 (F_2_) | 29 (F_3_) | 29 (F_4_) | 10 (F_5_) | 10 (F_6_) | 2 (OYT) | 1 (AYT) | - | - |
|  | Samba Mahsuri/MR 219 |  |  |  |  |  |  | 38 (F_1_) | 50 (F_2_) | 75 (F_3_) | 20 (F_4_) | 24 (F_5_) | 40 (F_6_) | 1 (OYT) | 1 (AYT) | - | - |
|  | Saro 5/Bunga mehu |  |  |  |  |  |  | 26 (F_1_) | 28 (F_2_) | 43 (F_3_) | 6 (F_4_) | 7 (F_5_) | 8 (F_6_) | 1 (OYT) | - | - | - |
|  | Swarna/UPL Ri 7 |  |  |  |  |  |  | 49 (F_1_) | 49 (F_2_) | 35 (F_3_) | 18 (F_4_) | 9 (F_5_) | 10 (F_6_) | 3 (OYT) | - | - | - |
|  | UPL Ri 7/IR 90266-B-228-1 |  |  |  |  |  |  | 75 (F_1_) | 44 (F_2_) | 17 (F_3_) | 14 (F_4_) | 11 (F_5_) | 6 (F_6_) | 4 (OYT) | 1 (AYT) | - | - |
|  | UPL Ri 7/IRRI 148 |  |  |  |  |  |  | 55 (F_1_) | 50 (F_2_) | 34 (F_3_) | 34 (F_4_) | 20 (F_5_) | 24 (F_6_) | 15 (OYT) | 1 (AYT) | - | - |
|  | WAB 878-6-37-4-4-P2-HB/IR 90265-B-551-1 |  |  |  |  |  |  | 58 (F_1_) | 51 (F_2_) | 5 (F_3_) | 1 (F_4_) | 1 (F_5_) | 6 (F_6_) | **-** | **-** | - | - |
|  | WAB 878-6-37-4-4-P2-HB/IR 90266-B-228-1 |  |  |  |  |  |  | 47 (F_1_) | 48 (F_2_) | 15 (F_3_) | 8 (F_4_) | 2 (F_5_) | 6 (F_6_) | **-** | **-** | - | - |
|  | HHZ 8-SAL 6-SAL 3-Y2/IR09L120 |  |  |  |  |  | 55 (F_1_) | 42 (F_2_) | 67 (F_3_) | 84 (F_4_) | 157 (F_5_) | 118 (F_6_) | 6 (OYT) | 4 (AYT) | 3 (AYT) | 2 (AYT) | 1 (AYT) |
|  | HHZ 8-SAL 6-SAL 3-Y2/IR09L204 |  |  |  |  |  | 50 (F_1_) | 39 (F_2_) | 62 (F_3_) | 49 (F_4_) | 71 (F_5_) | 78 (F_6_) | 18 (OYT) | 1 (AYT) | 1 (AYT) | - | - |
|  | HHZ 8-SAL 6-SAL 3-Y2/IR09L303 |  |  |  |  |  | 70 (F_1_) | 40 (F_2_) | 63 (F_3_) | 77 (F_4_) | 117 (F_5_) | 98 (F_6_) | 18 (OYT) | 4 (AYT) | 4 (AYT) | - | - |
|  | HHZ 8-SAL 6-SAL 3-Y2/IR09L336 |  |  |  |  |  | 46 (F_1_) | 42 (F_2_) | 39(F_3_) | 63 (F_4_) | 94 (F_5_) | 100 (F_6_) | 30 (OYT) | 1 (AYT) | - | - | - |
|  | HHZ 8-SAL 6-SAL 3-Y2/IR09L337 |  |  |  |  |  | 50 (F_1_) | 42 (F_2_) | 40 (F_3_) | 23 (F_4_) | 80 (F_5_) | 106 (F_6_) | 10 (OYT) | **-** | **-** | - | - |
|  | HHZ 8-SAL 6-SAL 3-Y2/Lakhon |  |  |  |  |  | 61 (F_1_) | 14 (F_2_) | 48 (F_3_) | 34 (F_4_) | 22 (F_5_) | 14 (F_6_) | 8 (OYT) | **-** | **-** | - | - |
|  | IR 67966-44-2-3/AGAMI M 1 |  |  |  |  |  | 38 (F_1_) | 44 (F_2_) | 59 (F_3_) | 53 (F_4_) | 28 (F_5_) | 12 (F_6_) | 6 (OYT) | **-** | **-** | - | - |
|  | IR05N412/IR09L336 |  |  |  |  |  | 68 (F_1_) | 33 (F_2_) | 73 (F_3_) | 80 (F_4_) | 286 (F_5_) | 106 (F_6_) | 32 (OYT) | 2 (AYT) | 1 (AYT) | - | - |
|  | IR06N155/IR09L120 |  |  |  |  |  | 55(F_1_) | 41 (F_2_) | 80 (F_3_) | 89 (F_4_) | 157 (F_5_) | 22 (F_6_) | 32 (OYT) | 4 (AYT) | 1 (AYT) | - | - |
|  | IR07N112/Arang |  |  |  |  |  | 48 (F_1_) | 34 (F_2_) | 35 (F_3_) | 80 (F_4_) | 45 (F_5_) | 23 (F_6_) | 18 (OYT) | **-** | - | - | - |
|  | IR08L181/Bulu Gendraj//IR 103569 |  |  |  |  |  | 50 (F_1_) | 44 (F_2_) | 30 (F_3_) | 99 (F_4_) | 59 (F_5_) | 24 (F_6_) | 20 (OYT) | 2 (AYT) | 1 (AYT) | - | - |
|  | IR09A228/IR09L179 |  |  |  |  |  | 55 (F_1_) | 43 (F_2_) | 69 (F_3_) | 56 (F_4_) | 77 (F_5_) | 12 (F_6_) | 6 (OYT) | **-** | **-** | - | - |
|  | IR09A228/Khilak |  |  |  |  |  | 48 (F_1_) | 41 (F_2_) | 23 (F_3_) | 166 (F_4_) | 107 (F_5_) | 18 (F_6_) | 6 (OYT) | **-** | **-** | - | - |
|  | IR09L179/B 1050 D-KN-1-1-1-1-3 |  |  |  |  |  | 41 (F_1_) | 35 (F_2_) | 31 (F_3_) | 196 (F_4_) | 135 (F_5_) | 36 (F_6_) | 24 (OYT) | 2 (AYT) | 1 (AYT) | - | - |
|  | IR09L179/Cicih gedu |  |  |  |  |  | 39(F_1_) | 33 (F_2_) | 24 (F_3_) | 115 (F_4_) | 80 (F_5_) | 15 (F_6_) | 6 (OYT) | **-** | **-** | - | - |
|  | IR09L204/Cicih gedu |  |  |  |  |  | 50 (F_1_) | 42 (F_2_) | 34 (F_3_) | 174 (F_4_) | 110 (F_5_) | 33 (F_6_) | 16 (OYT) | **-** | **-** | - | - |
|  | IR09L204/Ketan Merah |  |  |  |  |  | 52 (F_1_) | 35 (F_2_) | 10 (F_3_) | 152 (F_4_) | 73 (F_5_) | 12 (F_6_) | 4 (OYT) | **-** | **-** | - | - |
|  | IR09L303/Benong 130 |  |  |  |  |  | 45 (F_1_) | 40 (F_2_) | 43 (F_3_) | 39 (F_4_) | 18 (F_5_) | 36 (F_6_) | 5 (OYT) | 2 (AYT) | 2 (AYT) | - | - |
|  | IR09L303/Bunga Mehu |  |  |  |  |  | 66 (F_1_) | 57 (F_2_) | 42 (F_3_) | 39 (F_4_) | 6 (F_5_) | 44 (F_6_) | 1 (OYT) | 1 (AYT) | **-** | - | - |
|  | IR09L303/Glenteng |  |  |  |  |  | 78 (F_1_) | 58 (F_2_) | 21 (F_3_) | 78 (F_4_) | 31 (F_5_) | 34 (F_6_) | 1 (OYT) | 1 (AYT) | **-** | - | - |
|  | IR09L336/Bunga Mehu |  |  |  |  |  | 48 (F_1_) | 48 (F_2_) | 49 (F_3_) | 50 (F_4_) | 18 (F_5_) | 32 (F_6_) | 11 (OYT) | 1 (AYT) | 1 (AYT) | - | - |
|  | IR09L204/Ketan Merah |  |  |  |  |  | 50 (F_1_) | 35 (F_2_) | 10 (F_3_) | 59 (F_4_) | 32 (F_5_) | 16 (F_6_) | 4 (OYT) | - | - | - | - |
|  | IR09L337/Bunga Mehu |  |  |  |  |  | 67 (F_1_) | 52 (F_2_) | 55 (F_3_) | 45 (F_4_) | 20 (F_5_) | 34 (F_6_) | 42 (F_6_) | 40 (OYT) | 11 (AYT) | - | - |
|  | IR09L337/Cicih gedu |  |  |  |  |  | 75(F_1_) | 58 (F_2_) | 34 (F_3_) | 42 (F_4_) | 10 (F_5_) | 5 (F_6_) | 3 (F_6_) | 2 (OYT) | 1 (AYT) | - | - |
|  | IR09L342/Gropak gede 106 |  |  |  |  |  | 55 (F_1_) | 41 (F_2_) | 22 (F_3_) | 55 (F_4_) | 26 (F_5_) | 6 (F_6_) | 1 (OYT) | **-** | **-** | - | - |
|  | IR10A134/Lakhon |  |  |  |  |  | 40 (F_1_) | 10 (F_2_) | 16 (F_3_) | 92 (F_4_) | 44 (F_5_) | 16 (F_6_) | 11 (OYT) | **-** | **-** | - | - |
|  | IR10N108/IR09L337 |  |  |  |  |  | 55 (F_1_) | 45 (F_2_) | 86 (F_3_) | 14 (F_4_) | 5 (F_5_) | 8 (F_6_) | 1 (OYT) | **-** | **-** | - | - |
|  | PR 30245-10-414/IR09L179 |  |  |  |  |  | 50 (F_1_) | 42 (F_2_) | 34 (F_3_) | 112 (F_4_) | 58 (F_5_) | 11 (F_6_) | 3 (OYT) | **-** | **-** | - | - |
|  | PR 35805-B-9-2-3-2-3/IR09L337 |  |  |  |  |  | 40 (F_1_) | 10 (F_2_) | 82 (F_3_) | 98 (F_4_) | 52 (F_5_) | 104 (F_6_) | 1 (OYT) | **-** | **-** | - | - |
|  | PR 37139-3-1-3-1-2-1/IR09L303 |  |  |  |  |  | 62 (F_1_) | 44 (F_2_) | 71 (F_3_) | 68 (F_4_) | 54 (F_5_) | 108 (F_6_) | 23 (OYT) | 3 (AYT) | 2 (AYT) | - | - |
|  | TME 80518/Chao Med Nyay |  |  |  |  |  | 48 (F_1_) | 46 (F_2_) | 33 (F_3_) | 78 (F_4_) | 35 (F_5_) | 15 (F_6_) | 8 (OYT) | 1 (AYT) | 1 (AYT) | - | - |
|  | IR 65600-81-5-3-2/IR11L147 |  |  |  |  | 44 (F_1_) | 28 (F_2_) | 78 (F_3_) | 293 (F_4_) | 89 (F_5_) | 36 (F_6_) | 9 (OYT) | 3 (AYT) | 1 (AYT) | 1 (AYT) | - | - |
|  | IR 67962-84-2-2-2/IR08L216 |  |  |  |  | 40 (F_1_) | 71 (F_2_) | 72 (F_3_) | 266 (F_4_) | 159 (F_5_) | 40 (F_6_) | 21 (OYT) | 9 (AYT) | **-** | **-** | - | - |
|  | IR 67966-44-2-3/IR 87707-446-B-B-B |  |  |  |  | 50 (F_1_) | 66 (F_2_) | 66 (F_3_) | 160 (F_4_) | 119 (F_5_) | 19 (F_6_) | 10 (OYT) | 4 (AYT) | **-** | **-** | - | - |
|  | IR 67966-44-2-3/IR08L216 |  |  |  |  | 50 (F_1_) | 39 (F_2_) | 39 (F_3_) | 117 (F_4_) | 103 (F_5_) | 18 (F_6_) | 11 (OYT) | 5 (AYT) | **-** | **-** | - | - |
|  | IR 67966-44-2-3/Vandana |  |  |  |  | 50 (F_1_) | 46 (F_2_) | 46 (F_3_) | 115 (F_4_) | 118 (F_5_) | 16 (F_6_) | 8 (OYT) | 2 (AYT) | **-** | **-** | - | - |
|  | IR 72022-46-2-3-3-2/IR09L224 |  |  |  |  | 60 (F_1_) | 49 (F_2_) | 49 (F_3_) | 122 (F_4_) | 55 (F_5_) | 21 (F_6_) | 7 (OYT) | 1 (AYT) | **-** | **-** | - | - |
|  | IR 78875-176-B-2/IR 78875-207-B-3 | 65 (F_1_) | 55 (F_2_) | 161 (F_3_) | 101 (F_4_) | 47 (F_5_) | 30 (F_6_) | 6 (OYT) | 5 (AYT) | 2 (AYT) | 2 (AYT) | 2 (AYT) | 2 (AYT) | **-** | **-** | - | - |
|  | IR 81039-B-173-U 3-3/IR 81063-B-94-U 3-1 | 51 (F_1_) | 48 (F_2_) | 177 (F_3_) | 96 (F_4_) | 42 (F_5_) | 37 (F_6_) | 8 (OYT) | 2 (AYT) | 1 (AYT) | 1 (AYT) | 1 (AYT) | 1 (AYT) | **-** | **-** | - | - |
|  | IR 81896-B-B-182/Swarna | 50 (F_1_) | 75 (F_2_) | 99 (F_3_) | 63 (F_4_) | 36 (F_5_) | 22 (F_6_) | 6 (OYT) | 4 (AYT) | 1 (AYT) | 1 (AYT) | 1 (AYT) | 1 (AYT) | **-** | **-** | - | - |
|  | IR04A212/IR08L119 | 88 (F_1_) | 58 (F_2_) | 104 (F_3_) | 79 (F_4_) | 40 (F_5_) | 25 (F_6_) | 3 (AYT) | 3 (AYT) | 1 (AYT) | 1 (AYT) | 1 (AYT) | 1 (AYT) | **-** | **-** | - | - |
|  | IR05N173/IR09L272 |  | 50 (F_1_) | 62 (F_2_) | 165 (F_3_) | 161 (F_4_) | 41 (F_5_) | 21 (F_6_) | 8 (OYT) | 1 (AYT) | 1 (AYT) | 1 (AYT) | 1 (AYT) | **-** | **-** | - | - |
|  | IR05N173/IR10L149 |  | 55 (F_1_) | 46 (F_2_) | 40 (F_3_) | 36 (F_4_) | 29 (F_5_) | 59 (F_6_) | 7 (OYT) | 3 (AYT) | 3 (AYT) | 1 (AYT) | 1 (AYT) | **-** | **-** | - | - |
|  | IR06A144/IR 55419-04 | 60 (F_1_) | 82 (F_2_) | 46 (F_3_) | 34 (F_4_) | 55 (F_5_) | 37 (F_6_) | 11 (OYT) | 7 (AYT) | 4 (AYT) | 4 (AYT) | 2 (AYT) | 2 (AYT) | **-** | **-** | - | - |
|  | IR07L270/IR08L126 | 54 (F_1_) | 77 (F_2_) | 35 (F_3_) | 20 (F_4_) | 27 (F_5_) | 31 (F_6_) | 8 (OYT) | 10 (AYT) | 6 (AYT) | 2 (AYT) | 2 (AYT) | 1 (AYT) | **-** | **-** | - | - |
|  | IR07N112/IR11L186 | 50 (F_1_) | 59 (F_2_) | 39 (F_3_) | 24 (F_4_) | 40 (F_5_) | 26 (F_6_) | 5 (OYT) | 10 (AYT) | 7 (AYT) | 1 (AYT) | 1 (AYT) | 1 (AYT) | 1 (AYT) | 1 (AYT) | 1 (AYT) | - |
|  | IR08L217/IR08L183 | 50 (F_1_) | 60 (F_2_) | 28 (F_3_) | 15 (F_4_) | 33 (F_5_) | 16 (F_6_) | 8 (OYT) | 6 (AYT) | 2 (AYT) | 2 (AYT) | 2 (AYT) | 2 (AYT) | 1 (AYT) | **-** | **-** | - |
|  | IR08N120/IR10L182 | 44 (F_1_) | 55 (F_2_) | 29 (F_3_) | 22 (F_4_) | 38 (F_5_) | 22 (F_6_) | 7 (OYT) | 8 (AYT) | 4 (AYT) | 4 (AYT) | 4 (AYT) | 1 (AYT) | 1 (AYT) | 1 (AYT) | **-** | - |
|  | IR09L272/IR09L337 | 50 (F_1_) | 75 (F_2_) | 39 (F_3_) | 21(F_4_) | 44 (F_5_) | 38 (F_5_) | 20 (F_6_) | 10 (OYT) | 2 (AYT) | 2 (AYT) | 2 (AYT) | 2 (AYT) | 2 (AYT) | 1 (AYT) | **-** | - |
|  | IR09L272/IR10L105 |  | 55 (F_1_) | 53 (F_2_) | 38 (F_3_) | 23 (F_4_) | 32 (F_5_) | 24 (F_6_) | 15 (OYT) | 6 (AYT) | 6 (AYT) | 1 (AYT) | 1 (AYT) | 1 (AYT) | - | **-** | - |
|  | IR09L272/IR10L128 |  | 62 (F_1_) | 82 (F_2_) | 38 (F_3_) | 46 (F_4_) | 59 (F_5_) | 33 (F_6_) | 5 (OYT) | 12 (AYT) | 12 (AYT) | 1 (AYT) | 1 (AYT) | 1 (AYT) | 1 (AYT) | **-** | - |
|  | IR09L272/IR10L146 |  | 60 (F_1_) | 127 (F_2_) | 38 (F_3_) | 28 (F_4_) | 27 (F_5_) | 16 (F_6_) | 1 (OYT) | 4 (AYT) | 4 (AYT) | 1 (AYT) | 1 (AYT) | 1 (AYT) | 1 (AYT) | **-** | - |
|  | IR09L272/IR10L165 |  | 58 (F_1_) | 57 (F_2_) | 42 (F_3_) | 10 (F_4_) | 34 (F_5_) | 22 (F_6_) | 1 (OYT) | 3 (AYT) | 3 (AYT) | 1 (AYT) | 1 (AYT) | **-** | **-** | **-** | - |
|  | IR09L317/IR10L149 |  | 70 (F_1_) | 79 (F_2_) | 45 (F_3_) | 10 (F_4_) | 31 (F_5_) | 18 (F_6_) | 1 (OYT) | 1 (AYT) | 1 (AYT) | 1 (AYT) | 1 (AYT) | **-** | **-** | **-** | - |
|  | IR09L337/IR09L154 |  | 45 (F_1_) | 45 (F_2_) | 30 (F_3_) | 3 (F_4_) | 30 (F_5_) | 14 (F_6_) | 1 (OYT) | 2 (AYT) | 2 (AYT) | 1 (AYT) | 1 (AYT) | **-** | **-** | **-** | - |
|  | IR10F188/KHO487-4 |  | 40 (F_1_) | 64 (F_2_) | 44 (F_3_) | 8 (F_4_) | 37 (F_5_) | 11 (F_6_) | 2 (OYT) | 2 (AYT) | 2 (AYT) | 2 (AYT) | 2 (AYT) | **-** | **-** | **-** | - |
|  | IR10L149/IR10L165 |  | 60 (F_1_) | 59 (F_2_) | 41 (F_3_) | 10 (F_4_) | 25 (F_5_) | 18 (F_6_) | 1 (OYT) | 1 (AYT) | 1 (AYT) | 1 (AYT) | 1 (AYT) | **-** | **-** | **-** | - |
|  | IR10L411/IR11L261 |  |  |  |  | 50 (F_1_) | 235 (F_2_) | 113 (F_3_) | 461 (F_4_) | 251 (F_5_) | 133 (F_6_) | 95 (OYT) | 76 (AYT) | **-** | **-** | **-** | - |
|  | IR11L108/IR09N534 |  |  | 55 (F_1_) | 45 (F_2_) | 20 (F_3_) | 2 (F_4_) | 4 (F_5_) | 9 (F_6_) | 1 (OYT) | 1 (AYT) | 1 (AYT) | 1 (AYT) | **-** | **-** | **-** | - |
|  | IR11L152/Sabitri |  |  | 59 (F_1_) | 26 (F_2_) | 20 (F_3_) | 9 (F_4_) | 16 (F_5_) | 18 (F_6_) | 2 (OYT) | 1 (AYT) | 1 (AYT) | 1 (AYT) | **-** | **-** | **-** | - |
|  | IR11L186/IR09N534 |  |  | 50 (F_1_) | 28 (F_2_) | 20 (F_3_) | 3 (F_4_) | 9 (F_5_) | 12 (F_6_) | 2 (OYT) | 1 (AYT) | 1 (AYT) | 1 (AYT) | 1 (AYT) | 1 (AYT) | **-** | - |
|  | IR11L259/IR07A253 |  |  | 50 (F_1_) | 35 (F_2_) | 20 (F_3_) | 4 (F_4_) | 11 (F_5_) | 15 (F_6_) | 1 (OYT) | 1 (AYT) | 1 (AYT) | 1 (AYT) | **-** | - | **-** | - |
|  | IRRI 176/IR11L186 |  |  | 48 (F_1_) | 50 (F_2_) | 20 (F_3_) | 3 (F_4_) | 7 (F_5_) | 9 (F_6_) | 3 (OYT) | 3 (OYT) | 2 (AYT) | 2 (AYT) | 2 (AYT) | 2 (AYT) | 2 (AYT) | 1 (AYT) |
|  | IRRI 176/IR11L261 |  |  | 44 (F_1_) | 33 (F_2_) | 20 (F_3_) | 2 (F_4_) | 7 (F_5_) | 12 (F_6_) | 7 (OYT) | 7 (OYT) | 1 (AYT) | 1 (AYT) | **-** | **-** | **-** | **-** |
|  | WAB 878-6-37-4-4-P2-HB/IRRI 148 |  |  | 67 (F_1_) | 41 (F_2_) | 20 (F_3_) | 5 (F_2_) | 10 (F_3_) | 330 (F_4_) | 144 (F_5_) | 21 (F_6_) | 15 (OYT) | 6 (AYT) | **-** | **-** | **-** | **-** |
|  | WAB 878-6-37-4-4-P2-HB/Saro 5 |  |  | 70 (F_1_) | 28 (F_2_) | 20 (F_3_) | 6 (F_2_) | 8 (F_3_) | 117 (F_4_) | 27 (F_5_) | 8 (F_6_) | 8 (OYT) | 8 (AYT) | **-** | **-** | **-** | **-** |
| Triparent | IR 111249/IR 111250//BRRI Dhan 52/ |  |  |  |  |  |  |  |  | 121 (F_1_) | 51 (F_2_) | 91 (F_3_) | 25 (F_4_) | 35 (F_5_) | 7 (F_6_) | 2 (OYT) | **-** |
|  | IR 91648-B-1-B-3-1/Abhaya// IR11L101 |  |  |  |  |  |  |  |  | 82 (F_1_) | 44 (F_2_) | 74 (F_3_) | 12 (F_4_) | 15 (F_5_) | 3 (F_6_) | **-** | **-** |
|  | IR 91648-B-1-B-3-1/IRBB 23//IR 96322-34-223-B |  |  |  |  |  |  |  |  | 50 (F_1_) | 68 (F_2_) | 152 (F_3_) | 50 (F_4_) | 79 (F_5_) | 33 (F_6_) | 13 (OYT) | 3 (AYT) |
|  | IR09N538/Abhaya//IR09N538/IRBB60 (IR72920-1-44-4) |  |  |  |  |  |  |  |  | 68 (F_1_) | 28 (F_2_) | 27 (F_3_) | 17 (F_4_) | 31 (F_5_) | 30 (F_6_) | 13 (OYT) | 5 (AYT) |
|  | IR09N538/IR 93312-30-101-20-3-66-6//IR09N538/ IR11L101 |  |  |  |  |  |  |  |  | 76 (F_1_) | 59 (F_2_) | 29 (F_3_) | 12 (F_4_) | 17 (F_5_) | 17 (F_6_) | 6 (OYT) | 1 (AYT) |
|  | IR09N538/IR 97153-B-123-B//IR09N538/IR 97152-B-280-B |  |  |  |  |  |  |  |  | 133 (F_1_) | 56 (F_2_) | 23 (F_3_) | 6 (F_4_) | 6 (F_5_) | 2 (F_6_) | **-** | **-** |
|  | IR09N538/Tadukan//IR09N538/Rathu Heenati |  |  |  |  |  |  |  |  | 165 (F_1_) | 20 (F_2_) | 20 (F_3_) | 3 (F_4_) | 2 (F_5_) | 5 (F_6_) | 4 (OYT) | 2 (AYT) |
|  | IRRI 154/IR 94225-B-82-B//IRRI 154/IR 91648-B-32-B |  |  |  |  |  |  |  |  | 128(F_1_) | 60 (F_2_) | 92 (F_3_) | 27 (F_4_) | 36 (F_5_) | 10 (F_6_) | 7 (OYT) | 1 (AYT) |
|  | IRRI 154/IR 97153-B-123-B//IRRI 154/IR 97152-B-280-B |  |  |  |  |  |  |  |  | 108 (F_1_) | 61 (F_2_) | 71 (F_3_) | 31 (F_4_) | 37 (F_5_) | 8 (F_6_) | 5 (OYT) | 3 (AYT) |
|  | IRRI 154/IR11L101//IRRI 154/Rathu Heenati |  |  |  |  |  |  | 74 (F_1_) | 88 (F_2_) | 123 (F_3_) | 65 (F_4_) | 40 (F_5_) | 14 (F_6_) | 42 (F_5_) | 12 (F_6_) | 7 (OYT) | 1 (AYT) |
|  | IR09L303/Benong 130//IR09L342 |  |  |  |  |  |  | 50 (F_1_) | 45 (F_2_) | 42 (F_3_) | 38 (F_4_) | 20 (F_5_) | 56 (F_6_) | 6 (OYT) | 2 (AYT) | - | - |
|  | IR08L181/Bulu gendjah//IR 103569 |  |  |  |  |  | 86 (F_1_) | 55 (F_2_) | 30 (F_3_) | 25 (F_4_) | 18 (F_5_) | 3 (F_6_) | 24 (OYT) | 2 (AYT) | 2 (AYT) | - | - |
|  | IR09L120/C 7546 WH 2-2-1-1-9-2-3-2-1-2//IR09L120 |  |  |  |  |  | 67 (F_1_) | 50 (F_2_) | 43 (F_3_) | 47 (F_4_) | 58 (F_5_) | 40 (F_6_) | 3 (OYT) | 1 (AYT) | 1 (AYT) | - | - |
|  | IRRI 176/ IR11L226//IR 71700-247-1-1-2 |  |  |  |  |  |  | 31 (F_4_) | 69 (F_5_) | 129 (F_6_) | 7 (OYT) | 1 (AYT) | 1 (AYT) | **-** | **-** | - | - |
|  | IRRI 176/IR11L269//IR 71700-247-1-1-2 |  |  |  |  |  |  | 24 (F_4_) | 38 (F_5_) | 75 (F_6_) | 5 (OYT) | 2 (AYT) | 2 (AYT) | **-** | **-** | - | - |
|  | IR09L179/IR11L269//IR 71700-247-1-1-2 |  |  |  |  |  |  | 15 (F_4_) | 11 (F_5_) | 29 (F_6_) | 8 (OYT) | 2 (AYT) | 2 (AYT) | **-** | **-** | - | - |
|  | IRRI 154/Ma Zhan (RED)//IR08N194 |  |  |  |  |  |  | 11 (F_4_) | 15 (F_5_) | 30 (F_6_) | 3 (OYT) | 1 (AYT) | 1 (AYT) | **-** | **-** | - | - |
|  | IRRI 176/IR11L184//IR 71700-247-1-1-2 |  |  |  |  |  |  | 25 (F_4_) | 60 (F_5_) | 108 (F_6_) | 7 (OYT) | 1 (AYT) | 1 (AYT) | **-** | **-** | - | - |
|  | HHZ 8-SAL 6-SAL 3-Y2 /IR09L336//HHZ 8-SAL 6-SAL 3-Y2/IR09L204 |  |  |  |  |  |  | 64 (F_1_) | 197 (F_2_) | 49 (F_3_) | 24 (F_4_) | 21 (F_5_) | 30 F_6_ | 4 (OYT) | 3 (AYT) | 2 (AYT) | - |
|  | HHZ 8-SAL 6-SAL 3-Y2 /IR09L337//HHZ 8-SAL 6-SAL 3-Y2/IR09L303 |  |  |  |  |  |  | 100 (F_1_) | 180 (F_2_) | 67 (F_3_) | 33 (F_4_) | 41 (F_5_) | 58 F_6_ | 28 (OYT) | 13 (AYT) | 8 (AYT) | 2 (AYT) |
|  | PR 37951-3B-37-1-2/IR08L216//MR 219/IR08L216 |  |  |  |  |  |  | 100 (F_1_) | 160 (F_2_) | 42 (F_3_) | 18 (F_4_) | 15 (F_5_) | 10 F_6_ | 1 (OYT) | - | - | - |
| Quadraparent | HHZ 8-SAL 6-SAL 3-Y2/IR09L120//IR 86929-B-377-49-42/MTU 1010 |  |  |  |  |  |  | 45 (F_1_) | 196 (F_2_) | 31 (F_3_) | 6 (F_4_) | 7 (F_5_) | 6 F_6_ | **-** | **-** | - | - |
|  | IR 67966-44-2-3/IR 90265-B-551-1//IR 67966-44-2-3/IR 87707-446-B-B-B |  |  |  |  |  |  | 88 (F_1_) | 144 (F_2_) | 40 (F_3_) | 44 (F_4_) | 26 (F_5_) | 14 F_6_ | 3 (OYT) | 1 (AYT) | - | - |
|  | IR05N412/IR09L336//PR 37139-3-1-3-1-2-1/IR09L303 |  |  |  |  |  |  | 108 (F_1_) | 134 (F_2_) | 60 (F_3_) | 89 (F_4_) | 89 (F_5_) | 94 F_6_ | 16 (OYT) | 10 (AYT) | 5 (AYT) | 2 (AYT) |
|  | IR06N155/IR09L120//IR10N108/IR09L337 |  |  |  |  |  |  | 117 (F_1_) | 172 (F_2_) | 47 (F_3_) | 36 (F_4_) | 21(F_5_) | 22 F_6_ | 7 (OYT) | 1 (AYT) | 1 (AYT) | - |
|  | IR08N121/IR 90266-B-228-1// IR09L179/IR 88288-10-4-1-4 |  |  |  |  |  |  | 100 (F_1_) | 176 (F_2_) | 48 (F_3_) | 47 (F_4_) | 29 (F_5_) | 26 F_6_ | 5 (OYT) | 3 (AYT) | 1 (AYT) | - |
|  | IR10N237/IR08L216//IR09A228/IR09L179 |  |  |  |  |  |  | 63 (F_1_) | 143 (F_2_) | 31 (F_3_) | 11 (F_4_) | 11 (F_5_) | 10 F_6_ | 2 (OYT) | - | - | - |
|  | PR 30245-10-414/ IR09L179//PR 37866-1B-1-4/ IR08L216 |  |  |  |  |  |  | 72 (F_1_) | 180 (F_2_) | 45 (F_3_) | 49 (F_4_) | 36 (F_5_) | 16 F_6_ | 2 (OYT) | 1 (AYT) | - | - |
|  | PR 35805-B-9-2-3-2-3/IR09L337// IR06N119/IR09L336 |  |  |  |  |  |  | 53 (F_1_) | 175 (F_2_) | 48 (F_3_) | 34 (F_4_) | 31 (F_5_) | 34 F_6_ | 1 (OYT) | 2 (AYT) | - | - |
|  | IR09L179/Bea Balok Loas// IR09L120/B 1050D-KN-1-1-1-1-3 |  |  |  |  |  |  | 65 (F_1_) | 185 (F_2_) | 66 (F_3_) | 44 (F_4_) | 38 (F_6_) | 4 (OYT) | - | - | - | - |
|  | IR09L179/Benong 130//IR09L120/Beaoq Pendjalin |  |  |  |  |  |  | 68 (F_1_) | 162 (F_2_) | 45 (F_3_) | 27 (F_4_) | 8 (F_6_) | 4 (OYT) | - | - | - | - |
|  | IR09L204/B 1050 D-KN-1-1-1-1-3//IR09L303/Bea Balok Loas |  |  |  |  |  |  | 75 (F_1_) | 177 (F_2_) | 37 (F_3_) | 21 (F_4_) | 3 (F_6_) | 4 (OYT) | - | - | - | - |
|  | IR09L204/Bea Balok Loas//IR09L303/Benong 130 |  |  |  |  |  |  | 100 (F_1_) | 187 (F_2_) | 66 (F_3_) | 52 (F_4_) | 21 (F_6_) | 4 (OYT) | - | - | - | - |
|  | IR09L204/Benong 130//IR09L303/Bunga Mehu |  |  |  |  |  |  | 55 (F_1_) | 107 (F_2_) | 61 (F_3_) | 47 (F_4_) | 19 (F_6_) | 4 (OYT) | - | - | - | - |
|  | IR09L336/Benong 130//IR09L179/B 1050D-KN-1-1-1-1-3 |  |  |  |  |  |  | 101 (F_1_) | 133 (F_2_) | 80 (F_3_) | 55 (F_4_) | 18 (F_6_) | 1 (OYT) | - | - | - | - |
|  | IR09L337/Bea Balok Loas//IR09L336/Bea Balok Loas |  |  |  |  |  |  | 89 (F_1_) | 188 (F_2_) | 64 (F_3_) | 56 (F_4_) | 15 (F_6_) | 1 (OYT) | - | - | - | - |
|  | IR09L204/B 1050 D-KN-1-1-1-1-3//IR09L303/Bea Balok Loas |  |  |  |  |  |  | 60 (F_1_) | 152 (F_2_) | 60 (F_3_) | 49 (F_4_) | 21 (F_6_) | 4 (OYT) | - | - | - | - |
| Pentaparent | (NSIC Rc 222/WAB 880-1-27-9-2-P1-HB)/(IR87707-446-B-B-B/Dular)// (Dular/Kalinga 3) |  |  |  |  |  |  |  | 98 (F_1_) | 1,004 (F_2_) | 577 (F_3_) | 132 (F_4_) | 46 (F_5_) | 46 (F_6)_ | 2 (OYT) | - | - |
|  | NSIC Rc 222/ IR87707-446-B-B-B)/IR87707-446-B-B-B/IRRI148//Vandana/Kali Aus |  |  |  |  |  |  |  | 75 (F_1_) | 1,050 (F_2_) | 490 (F_3_) | 799 (F_4_) | 552 (F_5_) | 166 (F_6)_ | 125 (OYT) | 46 (AYT) | 25 (AYT) |
|  | (Swarna/IR87707-446-B-B)/(PSBRc 82/IRRI148)//(Vandana/IRRI148) |  |  |  |  |  |  |  | 109 (F_1_) | 685 (F_2_) | 585 (F_3_) | 209 (F_4_) | 42 (F_5_) | 43 (F_6)_ | 3 (OYT) | - | - |
|  | (PSBRc 82/WAB 880-1-27-9-2-P1-HB)/(PSBRc 82/ Dular)//(Kali Aus/Kalinga 3) |  |  |  |  |  |  |  |  | 144 (F_1_) | 566 (F_2_) | 358 (F_3_) | 39 (F_4_) | 39 F_5_ | 15 (F_6_) | 4 (OYT) | 2 (AYT) |
|  | P 2057-F4-88-3-1/IRAT 120// Suakoko///COL 1/M 312 A-74-2-8-8 |  |  |  |  |  | 50 (F_1_) | 544 (F_2_) | 312 (F_3_) | 39 (F_4_) | 18 (F_5_) | 15 (F_6_) | 1 (OYT) | 1 (AYT) | - | - | - |
| Hexaparent | Dular (acc. 32561)/IRRI 148// /IRRI 154/ UPLRi 7//IR87707-446-B-B-B/Kali Aus |  |  |  |  |  | 60 (F_1_) | 650 (F_2_) | 2,208 (F_3_) | 2,333 (F_4_) | 1,156 (F_5_) | 304 (F_6_) | 63 (OYT) | 63 (AYT) | 20 (AYT) | 12 (AYT) | 12 (AYT) |
|  | (Sahbhagi dhan/WAB 880-1-27-9-2-P1-HB) //(IRRI 123/ Dular) (acc. 32561)/// (Vandana/Kalinga 3) |  |  |  |  |  |  |  | 129 (F_1_) | 2,143 (F_2_) | 950 (F_3_) | 796 (F_4_) | 239 (F_5_) | 46 (F_6_) | 5 (OYT) | 2 (AYT) | 2 (AYT) |
|  | (Sahbhagi dhan/ IR87707-446-B-B)/(PSBRc 82/ IRRI148)// (Kali Aus/Kalinga 3) |  |  |  |  |  |  |  | 43 (F_1_) | 640 (F_2_) | 524 (F_3_) | 363 (F_4_) | 166 (F_5_) | 166 (F_6)_ | 33 (OYT) | 9 (AYT) | 7 (AYT) |
|  | (Sahbhagi dhan/UPLRi 7)/(PSBRc 82/Kali Aus)//(Vandana/IRRI148) |  |  |  |  |  |  |  | 58 (F_1_) | 1,016 (F_2_) | 746 (F_3_) | 640 (F_4_) | 394 (F_5_) | 434 (F_6)_ | 76 (OYT) | 27 (AYT) | 20 (AYT) |
|  | (Sambha Mahsuri/IR87707-446-B-B)/(UPLRi 7/IRRI148)//(Dular/Kali Aus) |  |  |  |  |  |  |  |  | 31 (F_1_) | 410 (F_2_) | 147 (F_3_) | 39 (F_4_) | 3 F_5_ | - | - | - |
